# Non-canonical transcriptional regulation of the poor prognostic factor UGT2B17 in chronic lymphocytic leukemic and normal B cells

**DOI:** 10.1186/s12885-024-12143-7

**Published:** 2024-04-02

**Authors:** Michèle Rouleau, Lyne Villeneuve, Eric P. Allain, Jules McCabe-Leroux, Sophie Tremblay, Flora Nguyen Van Long, Ashwini Uchil, Charles Joly-Beauparlant, Arnaud Droit, Chantal Guillemette

**Affiliations:** 1 Faculty of Pharmacy, Centre Hospitalier Universitaire de Québec Research Center - Université Laval (CRCHUQc- UL), Université Laval, Québec, QC Canada; 2 Cancer research center of Université Laval, Québec, Canada; 3 Molecular Genetics Laboratory, Vitalité Health Network, Dr. Georges-L.-Dumont University Hospital Center, Moncton, NB Canada; 4 CRCHUQc-UL and Faculty of Medicine, Université Laval, Québec, Québec, Canada; 5 Canada Research Chair in Pharmacogenomics, Faculty of Pharmacy, Université Laval, Québec, QC Canada

**Keywords:** Glycosyltransferase, Leukemic B cells, Alternative promoter, STAT3, NF-κB, Interferon regulation factors IRF

## Abstract

**Background:**

High expression of the glycosyltransferase UGT2B17 represents an independent adverse prognostic marker in chronic lymphocytic leukemia (CLL). It also constitutes a predictive marker for therapeutic response and a drug resistance mechanism. The key determinants driving expression of the *UGT2B17* gene in normal and leukemic B-cells remain undefined. The *UGT2B17* transcriptome is complex and is comprised of at least 10 alternative transcripts, identified by previous RNA-sequencing of liver and intestine. We hypothesized that the transcriptional program regulating *UGT2B17* in B-lymphocytes is distinct from the canonical expression previously characterized in the liver.

**Results:**

RNA-sequencing and genomics data revealed a specific genomic landscape at the *UGT2B17* locus in normal and leukemic B-cells. RNA-sequencing and quantitative PCR data indicated that the UGT2B17 enzyme is solely encoded by alternative transcripts expressed in CLL patient cells and not by the canonical transcript widely expressed in the liver and intestine. Chromatin accessible regions (ATAC-Seq) in CLL cells mapped with alternative promoters and non-coding exons, which may be derived from endogenous retrotransposon elements. By luciferase reporter assays, we identified key cis-regulatory STAT3, RELA and interferon regulatory factor (IRF) binding sequences driving the expression of UGT2B17 in lymphoblastoid and leukemic B-cells. Electrophoretic mobility shift assays and pharmacological inhibition demonstrated key roles for the CLL prosurvival transcription factors STAT3 and NF-κB in the leukemic expression of UGT2B17.

**Conclusions:**

UGT2B17 expression in B-CLL is driven by key regulators of CLL progression. Our data suggest that a NF-κB/STAT3/IRF/UGT2B17 axis may represent a novel B-cell pathway promoting disease progression and drug resistance.

**Supplementary Information:**

The online version contains supplementary material available at 10.1186/s12885-024-12143-7.

## Background

UGT2B17 is a metabolic enzyme that regulates the bioactivity of diverse endogenous molecules, including small signaling lipids (prostanglandin E_2_, leukotrienes), steroid hormones (androgens) as well as chemotherapeutic drugs such as vorinostat and fludarabine [1]. This enzyme is one of the 22 human UGT family members that catalyze the glycosylation of small lipophilic molecules. It uses primarily UDP-glucuronic acid as a sugar donor for the glucuronidation of its substrates. Whereas UGT2B17 and multiple other UGTs are expressed in the liver and the gastrointestinal tract, UGT2B17 is the main UGT expressed in B-cells [2–4]. Strikingly, higher B-cell expression of *UGT2B17* by nearly 2-fold is observed in 30–40% of patients with chronic lymphocytic leukemia (CLL), relative to normal B-cells. This elevated expression is associated with a more aggressive disease, shorter treatment-free and overall survival, independently of other prognostic markers, as shown in several cohorts [2, 3, 5–7]. Even in the more favorable prognostic subtype of CLL cases, defined by the mutational status of the immunoglobulin heavy chain variable region (IGHV) genes in B cells as mutated CLL (M-CLL), high *UGT2B17* predicts a poorer outcome relative to those with low *UGT2B17* levels [3, 6]. This highlights that high *UGT2B17* expression is clinically meaningful in both prognostic subgroups M-CLL and unmutated CLL (UM-CLL).

High UGT2B17 is further associated with a poor response to antileukemic treatments. Patients not responding to fludarabine-based treatments display higher *UGT2B17* expression, which is also induced in B-CLL cells by antileukemics treatments, namely by ibrutinib [2, 3]. The mechanisms by which UGT2B17 promotes progression and drug resistance are not fully characterized. Enzymatic inactivation of endogenous molecules such as prostaglandin E_2_ that impairs B-cell proliferation and of fludarabine that contributes to a largely reduced drug response, have been evidenced. However, functions unrelated to UGT2B17 enzymatic activity are also supported, given that leukemic cells expressing high levels of UGT2B17 are also resistant to anti-leukemics that are not UGT2B17 substrates [2, 3].

**Fig. 1:**
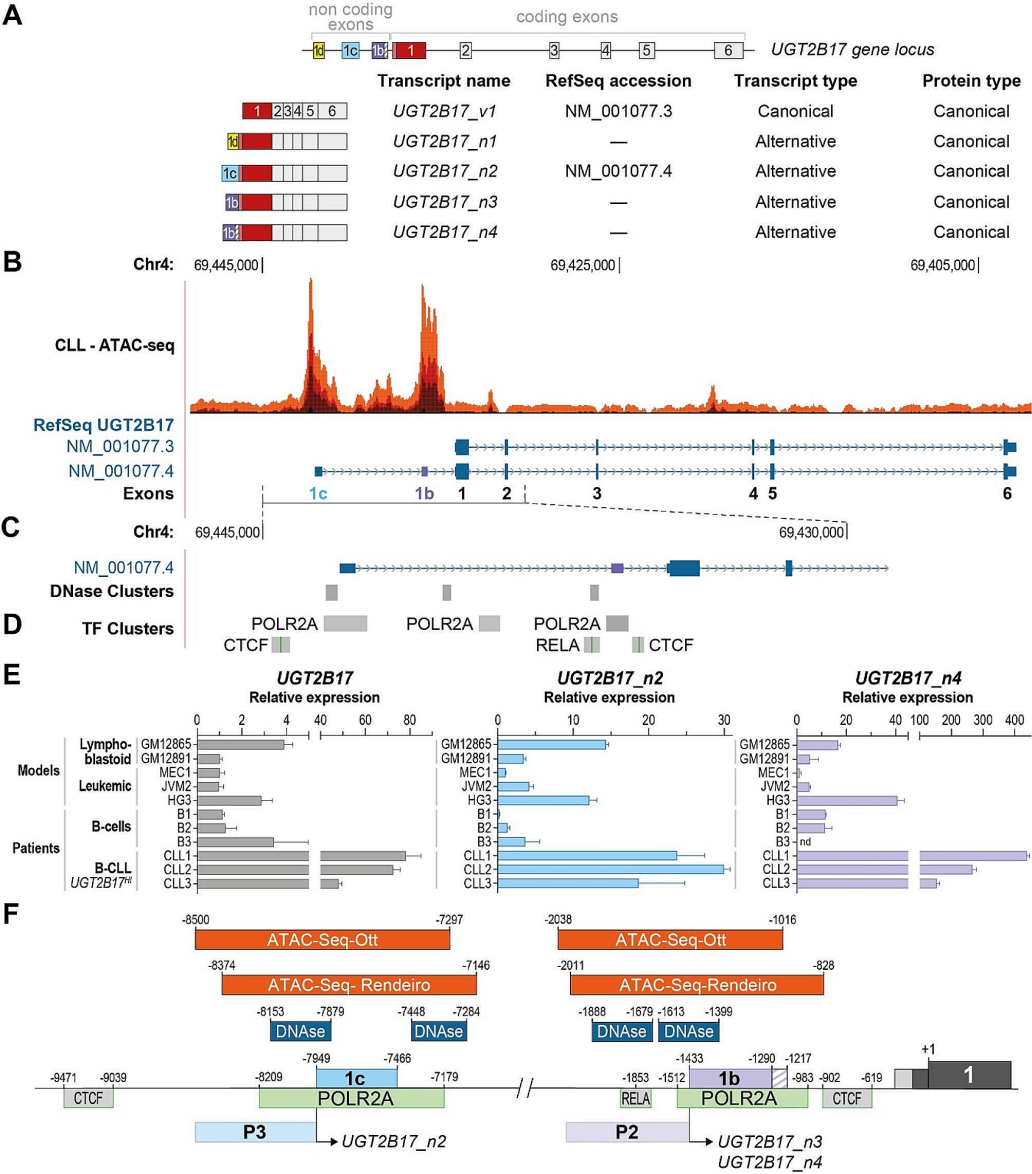
Chromatin landscape at the *UGT2B17* gene locus in lymphoblastoid cell models and CLL patient cells. **A.** The *UGT2B17* gene locus is comprised of six coding exons and alternative non-coding exons 1d, 1c and 1b. The canonical *UGT2B17_v1* and alternative *_n1*, *_n2*, *_n3*, and *_n4* transcripts all encode the full length UGT2B17 canonical enzyme. Transcripts *_n3* and *_n4* include the alternative exon 1b that is extended by 81 nucleotides on the 3’ side (purple hatched box) in transcript *_n4*. **B.** Transposase-accessible chromatin regions (ATAC-seq) at the *UGT2B17* gene locus across a cohort of CLL patients (*n* = 88 [16]). The canonical transcript *UGT2B17_v1* (RefSeq accession NM_001077.3) and the most recent RefSeq *UGT2B17* gene annotation NM_001077.4 encoding *UGT2B17_n2* are shown (Annotation Release NCBI Homo sapiens 105.20220307). Note that the *UGT2B17* locus is on the reverse strand/orientation. **C.** DNAse hypersensitive sites in ENCODE (V3; *n* = 125 cell models) and **D.** Transcription factor-bound chromatin (ChIP-seq clusters (*n* = 161 factors) from ENCODE with Factorbook Motifs) at the *UGT2B17* 5’UTR gene locus detected in lymphoblastoid cell models; Data in panels B-D was obtained from the UCSC browser (http://genome.ucsc.edu) [17] version GRCh37/hg19 accessed in January 2023. The position of exon 1b, not included in the current public gene annotation, is indicated by a purple box. **E.** Relative expression of total *UGT2B17*, *UGT2B17_n2* and *UGT2B17_n4* transcripts assessed by RT-qPCR in lymphoblastoid and B-CLL cell models previously studied to demonstrate UGT2B17 oncogenic functions [2, 3, 5, 18], and in B-cells from healthy individuals and CLL patients. Note that CLL patients expressing high levels of UGT2B17 (UGT2B17^HI^) were screened. **F.** Chromatin features at the *UGT2B17* gene locus identify P3 and P2 regions as the main transcriptional regulatory regions in CLL and lymphoblastoid cell models whereas the canonical *UGT2B17_v1* expression is undetected [2]. Coordinates are relative to the UGT2B17 translation start site (+ 1) according to the data on the UCSC browser (GRCh37/hg19). The features of the genomic DNA sequence at the *UGT2B17* locus are detailed in Supplementary data 1, Supplementary file 1

Our previous work revealed that the regulation of UGT2B17 enzyme expression is complex. At least 10 transcripts are expressed from the *UGT2B17* gene locus (Fig. 1A) [2, 8, 9]. The cis and trans-acting determinants of the canonical *UGT2B17* expression have been studied mostly in the liver in which the transcription factor forkhead box protein A1 (FOXA1) constitutes a critical regulator [10–12]. The FOXA1 binding site lies proximal to the UGT2B17 canonical exon 1, and a single nucleotide polymorphism within this regulatory element drastically impacts the hepatic expression of UGT2B17 [10, 11]. Alternative transcripts represent only a minor fraction of *UGT2B17* expression in the liver and the intestine, as recently uncovered [9, 13]. The regulation of their expression remains uncharacterized. Other regulatory elements include miRNAs that have been shown to modulate UGT2B17 levels in the prostate [14, 15]. Our recent work has revealed an alternative UGT2B17 expression profile in extra-hepatic tissues. In prostate cancer and leukemic cells, alternative transcripts rather than the canonical transcript drive UGT2B17 enzyme expression [2, 13]. These alternative transcripts include additional non-coding exons, but still encode the full-length canonical enzyme (Fig. 1A) [8]. Given the prooncogenic and drug-resistance functions of UGT2B17 in CLL, we sought to characterize the determinants of its expression in B-cells.

## Results

### Expression of UGT2B17 in normal and leukemic B-cells is driven by non-canonical/alternative transcripts

Among the 10 *UGT2B17* transcripts, five encode the full length UGT2B17 enzyme (Fig. 1A) [8]. The alternative *n1*, *n2*, *n3* and *n4* variants diverge from the canonical *v1* transcript by mutually exclusive alternative exons 1d, 1c and 1b that we described previously [8, 9]. RNA-sequencing indicated that the canonical *UGT2B17_v1* transcript was not expressed in leukemic cells from CLL patients and in the leukemic cell models MEC1 and JVM2 [2]. It suggested that the UGT2B17 enzyme was encoded by the alternative transcripts *n2*, *n3* and *n4*, which include the alternative exons 1c and 1b.

Publicly available chromatin accessibility data generated by Assay for Transposase-Accessible Chromatin using sequencing (ATAC-Seq) in two small cohorts of B-cells from CLL patients [16, 19] indicated that the genomic regulatory regions upstream of exons 1c and 1b lie in an open chromatin state, supporting that active transcription is ongoing at these alternative UGT2B17 exons in CLL patient’s cells of both M-CLL and UM-CLL prognostic subgroups (Fig. 1B; Fig. S1 and Fig. S2, Supplementary file 1). ChIPmentation chromatin mapping of acetylated H3K27 signals, absence of the repressive H3K27me3 epigenetic marks, and RNA-seq reads mapping to the genomic regions of exons 1c and 1b in the same CLL patient’s cells collectively provide evidence of active transcription at these specific genomic regions (see Fig. S2, Supplementary file 1). DNAse hypersensitive regions, indicative of regulatory/promoter regions, which have been gathered on multiple cell models by the ENCODE project, also revealed an open chromatin state at the genomic exon 1c and 1b sequences in B-lymphoblastoïd cell models. Further indicative of active transcription at these genomic regions, the ChIP-Seq data of the ENCODE project revealed the presence of RNA polymerase II (POL2A) encompassing exon 1c and exon 1b in B-lymphocyte cell models, as well as the chromatin insulator CTCF, facilitating enhancer recruitment [20, 21], near exons 1c and 1b in the same cell models (Fig. 1C, D). We also evidenced the expression of *UGT2B17_n2* and *UGT2B17_n4* transcripts in B-cells from healthy and CLL patients as well as in B-cell and B-CLL cell models using RT-qPCR with specific primers for the alternative transcripts (Fig. 1E), a finding also supported by the full-length sequencing of the *UGT2B17_n2* transcript by the PacBio approach in the B-lymphoblastoïd cell model GM12891 [2]. In all, the chromatin landscape at the *UGT2B17* gene locus suggested a B-cell regulation of UGT2B17 expression divergent from that in drug metabolizing tissues, namely liver and intestine (Fig. 1F).

### NF-κB, STAT3, and IRF1 are key regulators *UGT2B17* expression in B-cells

**Fig. 2:**
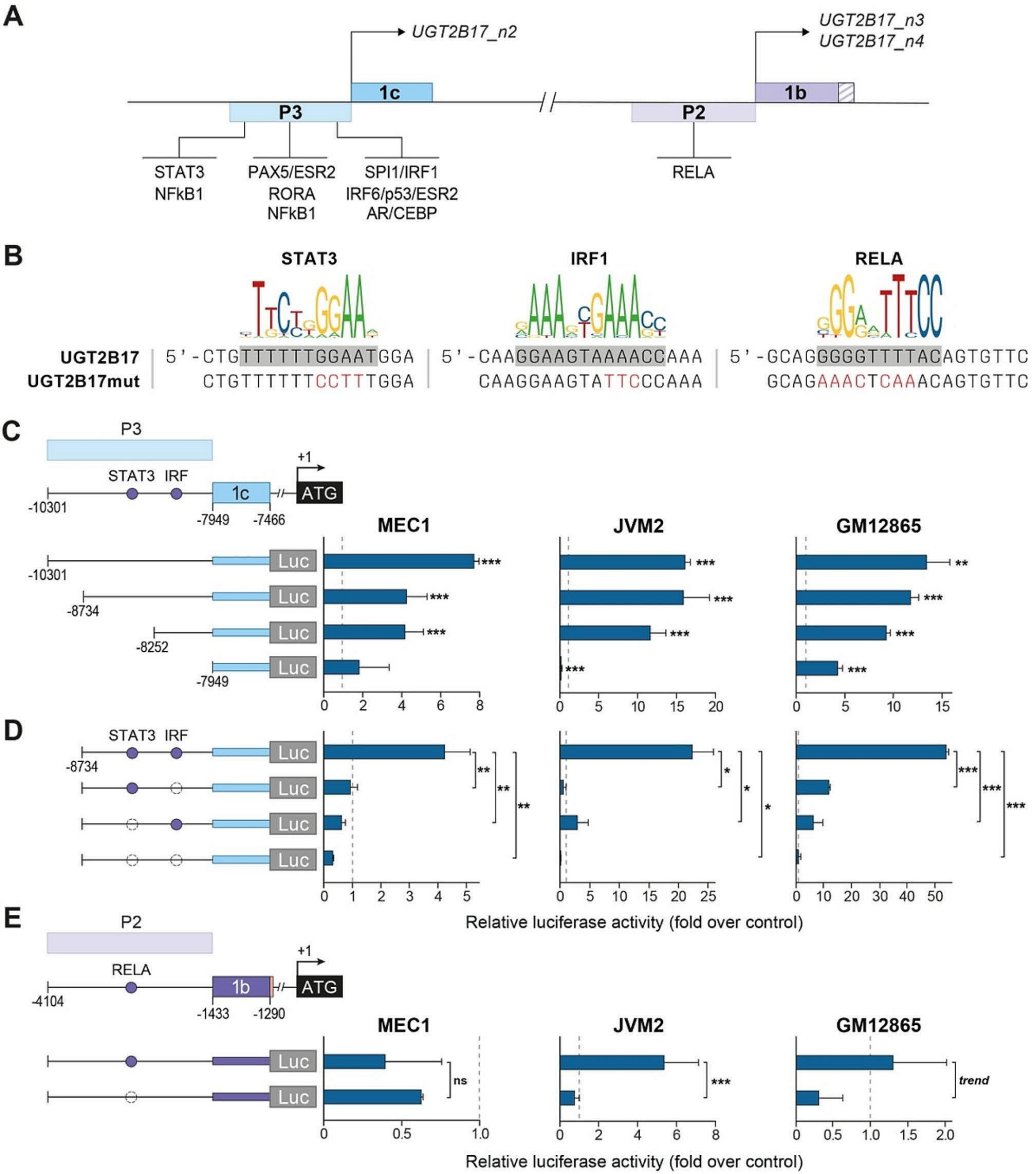
Cis-regulatory regions in the P3 and P2 promoter of UGT2B17 alt. transcripts. **(A)** Transcription factor (TF) binding sites predicted by JASPAR [22] in the P3 and P2 promoter regions. STAT3: Signal transducer and activator of transcription 3; NF-κB1: nuclear factor kappa B subunit 1 (p105); PAX5: paired box 5; ESR2: estrogen receptor 2; RORA/B: RAR related orphan receptor A/B; RELA: nuclear factor NF-kappa-B p65; SPI1: transcription factor PU.1; IRF1: Interferon regulatory factor 1; AR: androgen receptor; CEBP: CCAAT/enhancer binding protein. **(B)** Sequences of the consensus binding sites for STAT3 (MA0144.2), IRF1 (MA0050.1) and RELA (MA0107.1) are shown along with the corresponding sequence in the *UGT2B17* P3 or P2 region. Mutations of TF binding motifs for luciferase assays and EMSA (UGT2B17mut) are shown in red. **(C)** Luciferase (Luc) gene reporter assays with serial deletions of the P3 promoter region. Luciferase activity relative to the pGL3 empty vector (dashed line) is given. **(D)** Luc assays for P3 promoter activity without (filled circles) or with (open circles) mutated STAT3 and IRF1 binding sites. **(E)** Luc assays for P2 promoter activity without (filled circles) or with (open circles) mutagenesis of the RELA binding site. In D-E, the luciferase activity relative to the unmutated sequence is shown. Luciferase assays were conducted four times in triplicates. *, *P* ≤ 0.05; **, *P* ≤ 0.01; ***, *P* ≤ 0.001. Coordinates are relative to the UGT2B17 translation start site (+ 1)

We next sought to identify drivers of *UGT2B17* expression in B-cells, likely distinct from those previously identified to regulate the hepatic and intestine expression of the UGT2B17 enzyme, mainly encoded by the canonical *v1* transcript [10–13]. An *in silico* prediction of transcription factor (TF) binding sites with JASPAR in regions upstream of exon 1c (P3) and exon 1b (P2) revealed a number of potential TF binding sites (Fig. 2A,B) that were investigated with luciferase assays in the leukemic MEC1 and JVM2 and B-lymphoblastoid GM12865 cell models.

The first series of luciferase assays with serial deletions of the 10 kbp region upstream of exon 1c identified that the main regulatory region is located in the 784 bp sequence upstream of exon 1c (Fig. 2C). This sequence is rich in predicted TF binding sites, which were screened by mutagenesis to identify critical sequences. Mutagenesis of the predicted NF-kB1, PAX5, ESR2, RORA, IRF6, p53 and STAT3_− 8409_ binding sites did not affect expression of the luciferase gene (Fig. S3, Supplementary file 1). Mutagenesis of the predicted AR/CEBP binding site enhanced luciferase expression in GM12865 cells, suggesting that these factors might act as repressors, an observation that was not investigated further. By contrast, point mutations introduced in the predicted IRF1 and STAT3_− 8538_ binding sites nearly abolished luciferase expression in each tested cell model (Fig. 2B,D).

In the P2 region upstream of exon 1b, a binding site for the NF-κB subunit RELA was predicted at position − 1853 by JASPAR. RELA binding to this site has been previously shown by the ENCODE project using ChIP-Seq in two B-lymphoblastoid cell lines treated with TNF-α (Fig. 1D). Point mutations introduced in the RELA binding sequence reduced luciferase expression in JVM2 and GM12865 (Fig. 2B,E), supporting that NF-κB regulates expression of *UGT2B17_n3/n4* in B-cells.

### Regulation of UGT2B17 expression by NF-κB and STAT3 in leukemic cells

**Fig. 3:**
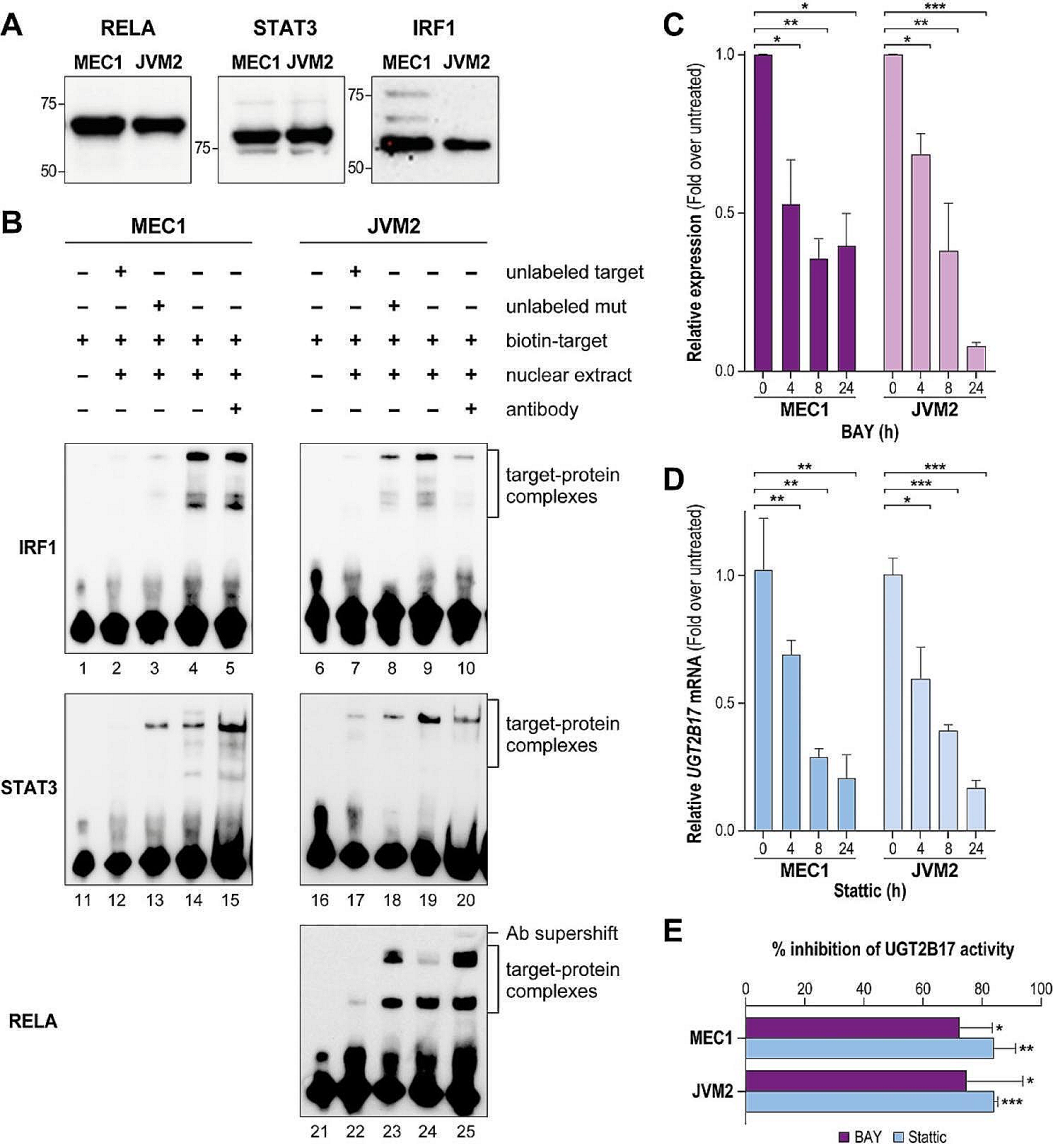
NF-κB and STAT3 regulate UGT2B17 expression in leukemic cell models. **(A)** Immunoblot detection of RELA, STAT3 and IRF1 in the MEC1 and JVM2 cell models. Full-length blots are presented in figure S5, additional file 1. **(B)** Electrophoretic mobility shift assays (EMSA) with biotin labeled oligonucleotides of IRF1, STAT3, or RELA (NF-κB) binding sequences and nuclear extracts of MEC1 and JVM2. The shift of the biotin labeled probes (lanes 4, 9; 14, 19; 24) is impaired by incubation with an excess unlabeled probe (lanes 2, 7; 12, 17; 22) but not with an excess of unlabeled mutated probe (lanes 3, 8; 13, 18; 23). The presence of the RELA antibody produced a supershifted complex (lanes 25). EMSA experiments were conducted twice. Full-length blots and replicates are presented in Figures S6 and S7, Supplementary file 1. **(C)** inhibition of *UGT2B17* mRNA expression by the NF-κB inhibitor BAY 11-7082 in leukemic cell models. **(D)** Inhibition of *UGT2B17* mRNA expression by the STAT3 inhibitor Stattic in leukemic cell models. **(E)** Inhibition of UGT2B17 protein expression by BAY 11-7082 and Stattic. Protein expression was measured by enzymatic assays after a 24 h-treatment with inhibitors, as described in the Methods section, and is expressed relative to the activity in untreated cells. Two independent qPCR and enzymatic assay experiments were conducted in triplicates. *, *P* ≤ 0.05; **, *P* ≤ 0.01; ***, *P* ≤ 0.001

The transcription factors RELA, STAT3 and IRF1 are well expressed in B-CLL cells and the leukemic cell models MEC1 and JVM2 (Fig. 3A). To demonstrate their interaction with the cis-regulatory sequences at P2 and P3 promoters, DNA/protein complexes were examined by electrophoretic mobility shift assays (EMSA) using nuclear extracts from the leukemic MEC1 and JVM2 cells. The biotin-labeled probe bearing the RELA binding sequence (P2 promoter) formed three complexes (Fig. 3B, lane 24; Fig. S6). An excess of the unlabeled probe competed with the formation of complexes but not the probe bearing mutations disrupting the RELA binding site (Fig. 3B, lanes 22–23; Fig. S6). Inclusion of a RELA antibody generated a supershifted complex, supporting that RELA is part of the DNA/protein complexes (Fig. 3B, lane 25; Fig. S6). Several DNA/protein complexes were also formed with probes bearing the STAT3 and IRF1 binding sequences of the P3 promoter (Fig. 3B, lanes 4, 9, 14, 19). Unlabeled probes competed with the formation of DNA/protein complexes (Fig. 3B, lanes 2, 7, 12, 17) whereas mutated unlabeled probes did not (Fig. 3B, lanes 3, 8, 13, 18). Supershifted complexes were not detected when TF specific antibodies were included in the assays. This observation remains to be explained, and might be due to protein-TF interactions masking antibody binding sites.

Further evidence that NF-κB and STAT3 are involved in the expression of UGT2B17 in leukemic cells was also demonstrated by their pharmacological inhibition with BAY 11-7082 and Stattic, respectively. Each inhibitor impaired *UGT2B17* mRNA expression (Fig. 3C-D), and considerably reduced the UGT2B17 enzyme activity by 72–84% (Fig. 3E), both in MEC1 and JVM2 cells.

### Associations between the expression of *UGT2B17* and the transcription factors NF-κB, STAT3, and IRF in CLL patient cells

**Fig. 4:**
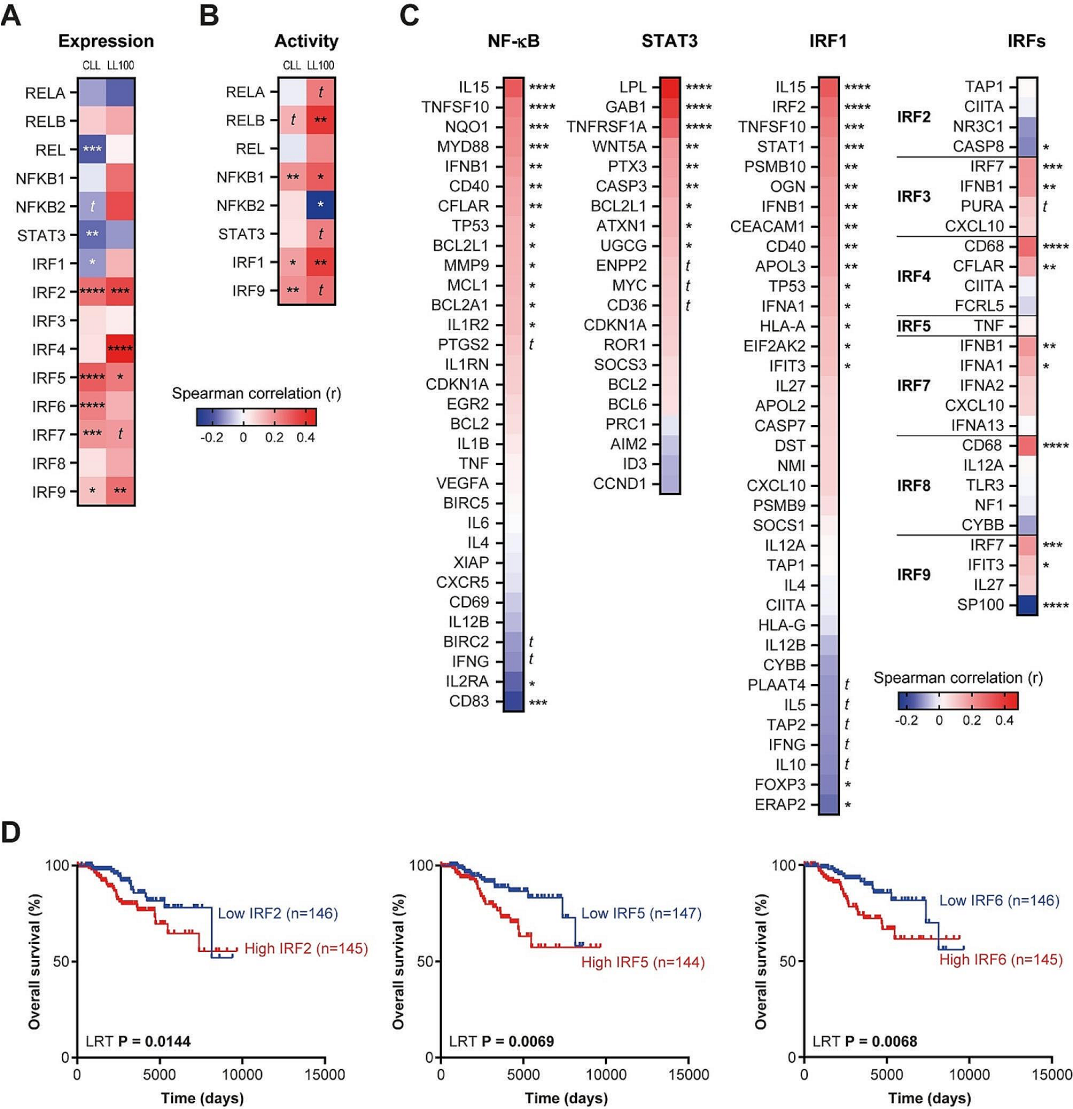
Correlations between *UGT2B17* expression and regulatory transcription factors (TFs) in the ICGC CLL cohort and LL100 preclinical leukemic cells. (A) Correlation of *UGT2B17* expression with the expression of regulatory TF in CLL cases of the ICGC cohort (*n* = 294) and in the lymphoma and leukemic cell models of the LL100 collection. (B) Correlation of *UGT2B17* expression with TF activity according to DoRothEA regulons in the CLL/ICGC cohort and the LL100 collection. (C) Correlation of *UGT2B17* expression with the expression of NF-κB, STAT3 and IRFs target genes relevant to CLL in the ICGC cohort. (D) The elevated expression of *IRF2*, *IRF5* and *IRF6* are associated with poor overall survival in CLL patients of the ICGC cohort, dichotomized on the median expression of each TF. LRT: log-rank test. *, *P* ≤ 0.05; **, *P* ≤ 0.01; ***, *P* ≤ 0.001; ****, *P* ≤ 0.0001; *t*, trend

In the International Cancer Genome Consortium (ICGC) cohort of CLL patients, the expression of *UGT2B17* correlated weakly with the expression of NF-κB subunits and *STAT3* (Fig. 4A), whereas it correlated significantly with the transcriptional activity of NF-κB and STAT3, based on the expression of known gene targets for these TFs, in any tissues, as quantified by DoRothEA [23] (Fig. 4B). With a focus on targets relevant to CLL, the expression of *UGT2B17* correlated well with that of multiple target genes of NF-κB and STAT3, including the NF-κB targets *IL15*, *TNFSF10*, *NQO1*, *MYD88*, *CD40* and *CFLAR* (encoding cFLIP), and the STAT3 targets lipoprotein lipase (*LPL*), *GAB1*, *TNRRFS1A* and *WNT5A*, among others (Fig. 4C). The expression of *IRF1* did not correlate positively with *UGT2B17* expression (Fig. 4A), but its transcriptional activity quantified by DoRothEA did correlate significantly with *UGT2B17* expression, and correlated well with a considerable number of IRF1 gene targets in the ICGC CLL cohort (Fig. 4B,C). Given that IRF1 is part of a family of nine IRF factors that bind to similar consensus sequences, we explored the correlations with the expression of the other IRFs and their documented target genes. A significant positive correlation was observed between *UGT2B17* and *IRF2*, *IRF5*, *IRF6*, *IRF7*, and *IRF9* expression, as well as with several of their targets (Fig. 4C). The relevance of IRFs in CLL is supported by the observation that the high expression of several IRFs (*IRF2*, *IRF5* and *IRF6*) in B-CLL patient cells is associated with poor survival outcome (Log-rank test (LRT) < 0.015) (Fig. 4D). Using expression data collected from the LL100 collection composed of 100 lymphocytic and leukemic cell lines from both lymphoid and myeloid lineages, we gathered further evidence that the expression of *UGT2B17* is well correlated with the expression and activity of TFs and target genes (Fig. 4A-C; Fig. S4, Supplementary file 1).

## Discussion

The expression of UGT2B17 is primarily regulated at the transcription level. While hepatic (liver) cell models have provided valuable insights into UGT2B17 regulation, our work demonstrates that its expression and regulation differ in normal and leukemic B-cells as well as in B-cell models. It involves transcription factors that are critical for B-cell survival and for pro-oncogenic events.

**Fig. 5:**
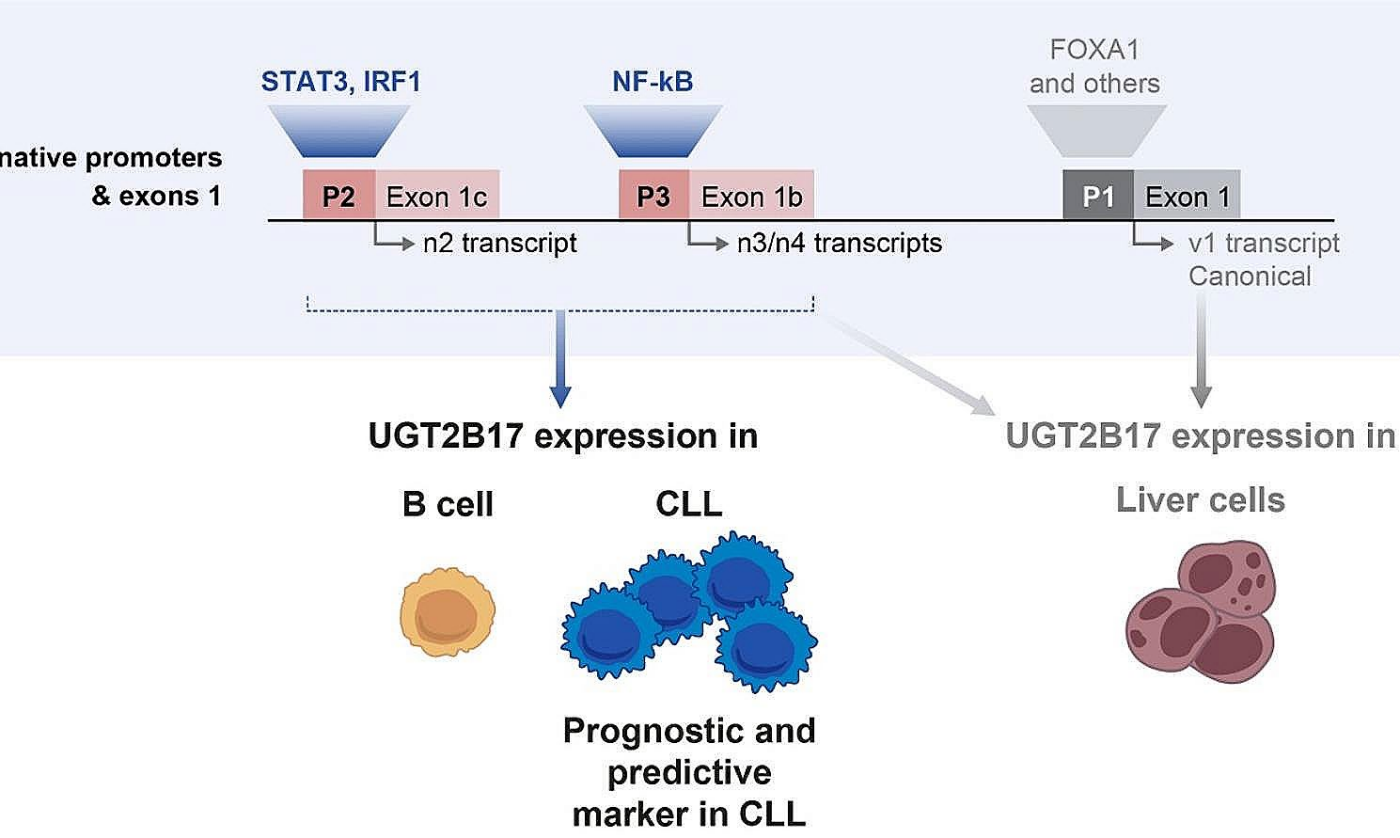
Schematic overview of the regulatory mechanisms governing the expression of UGT2B17 in B-CLL and normal cells. The UGT2B17 enzyme expression is solely derived from alternative transcripts n2, n3, n4 in B-CLL and normal B cells and is regulated by STAT3, IRF1 and NF-κB. In the liver, UGT2B17 enzyme expression is driven by the canonical transcript v1 and regulated by FOXA1, HNF1 and other transcription factors, whereas alternative transcripts constitute only a minor fraction of the hepatic *UGT2B17* transcriptome

Previous studies of hepatic and prostate cancer cell models focused on the canonical promoter located proximal to the first coding exon 1 of the *UGT2B17* gene locus and identified several response elements including FOXA1 and hepatocyte nuclear factor 1 as transcriptional regulators for the hepatic and prostate expression [10–12, 24, 25]. The determinants of UGT2B17 expression in B-cells had not been determined. They are relevant to identify since they can provide valuable insight into the underlying mechanisms of UGT2B17 expression as a prognostic and predictive marker in CLL, and in B-cells and dendritic cells previously recognized to produce UGT2B17 minor antigens responsible for cases of graft-vs-host diseases [26, 27]. Our findings provide strong evidence that *UGT2B17* expression in B-cells is driven solely by alternative promoters located over 1400 and 8000 bp upstream of the ATG translational start site, and that transcripts encoding the UGT2B17 enzyme include mutually exclusive alternative exons. We further evidenced that key TFs implicated in hematological malignancies and oncogenic signaling pathways in B-cells, NF-kB, STAT3 and IRF1, are important regulators of UGT2B17 expression in B-cells (Fig. 5).

The complexity of the *UGT2B17* transcriptome has recently been recognized [2, 8, 13]. In B-cells and B-CLL, it is composed of three alternative transcripts, namely *UGT2B17_n2*, *_n3* and *_n4* that include alternative, non-coding and mutually exclusive 5’ exons 1c and 1b [2]. The main canonical *UGT2B17_v1* transcript expressed in the liver is not expressed in B-cells [2, 9, 13], reinforcing the relevance of our findings. Our *in silico* and molecular analysis of cis-regulatory sequences near alternative exons 1c and 1b revealed binding sites for STAT3, IRF1 and NF-κB. Pharmacological inhibition further supported the regulatory role of STAT3 and NF-κB in the expression of *UGT2B17* in B-CLL cells. This is consistent with the known survival advantage conferred by STAT3 activation in CLL namely by upregulating expression of anti-apoptotic genes, enhancing the B-cell receptor (BCR) signaling, promoting interactions with the microenvironment, modulating the immune response and contributing to immune evasion [28, 29]. STAT3 is constitutively activated by phosphorylation in CLL [29, 30], drives the expression of genes involved in CLL progression and cell survival, as well as CLL poor prognosis markers such as LPL, ROR1 and WNT5a [31–36], and UGT2B17, as we report here. Similarly, NF-κB activation also promotes proliferation and survival of normal and leukemic B-cells and plays a role in chemotherapies and targeted drug resistance [31, 37, 38], and is one mediator of STAT3 activation in CLL [31, 39]. For example, a NF-κB/JAK2/STAT3 signaling axis triggered by IFNγ and IL-6 cytokines constitutes complex pro-survival signals that promote CLL progression by stimulating cell proliferation and inhibiting apoptosis [28–30, 39, 40]. The role of STAT3 and NF-κB in the regulation of the poor prognosis and proliferation-promoting UGT2B17 is thus well in line with its oncogenic functions, and warrants further investigation. By contrast, the expression of FOXA1, important for the hepatic expression of *UGT2B17_v1* [11, 13], was detected in less than 3% of CLL patients B-cells of the ICGC cohort, making it unlikely to regulate *UGT2B17* expression in CLL. Several gene targets of NF-kB and STAT3 significantly correlated with UGT2B17 expression consistent with a regulation of *UGT2B17* by this signaling axis in leukemic cells of CLL patients. The dysregulation of IRF proteins in CLL also suggests their involvement in disease pathogenesis and progression. There are nine Interferon Regulatory Factors (IRFs) that share a related DNA binding motif and act as homo and heterodimers that modulate their regulatory functions [41]. A co-regulation by IRF1 and/or other members of the IRF family might contribute to *UGT2B17* expression. *UGT2B17* expression correlated with the expression of several IRFs, with IRF1 and IRF9 transcriptional activity, as well as several gene targets of IRFs, including *IRF4* associated with poor CLL survival namely by controlling responsiveness to BCR stimulation in CLL [42]. In our recent investigations, we evidenced that the UGT2B17 protein is a novel constituent of BCR signalosome also connected with microenvironmental signaling [18].

The genomic region containing *UGT2B17* alternative exons and regulatory sequences may originate from transposable elements (TEs) of the human endogenous retrovirus (HERV/ERV1) type [43–45]. The *UGT2B17* genomic organization shares some similarity with the *CD5* gene that encodes transcripts with alternative exons 1 in B-cells and B-CLL cells affecting CD5-mediated signaling pathways, owing to a HERV-E sequence upstream of the canonical first exon [46]. TEs, acquired through ancient retroviral infections, are rich in transcriptional regulatory sequences [47–49]. By epigenetic mechanisms, they are typically silenced or repressed in normal cells but reactivated in cancer cells where they trigger oncogenic gene expression that promote the initiation and progression of human cancers, including CLL and other hematological malignancies [50–53]. Although we could show that UGT2B17 is expressed in normal B-cells, its higher expression in B-CLL patients’ cells, and further enhanced in CLL cases with poor prognosis, may involve such a mechanism.

The activation of endogenous retroelements are also increasingly implicated in the immune surveillance of human cancers [54], and other recent studies suggested that some transcribed and translated TEs may constitute tumor-specific antigens [51, 55]. In this context, targeting the epigenetic landscape of TE genomic regions could constitute a novel therapeutic approach for several malignancies and other diseases [49, 51, 55, 56]. Three unique UGT2B17 peptides constitute minor human leukocyte antigens responsible for graft-vs-host diseases (GVHD) after allogeneic bone marrow transplant in donor-recipient mismatch for the *UGT2B17* gene [26, 27]. It remains to be examined whether the expression of UGT2B17 in B-CLL cells could provide leukemic cell antigens targetable by an immunological approach.

In conclusion, this study evidenced a B-cell specific regulation of UGT2B17 expression characterized by alternative promoters regulated by pro-survival transcription factors involved in CLL progression and patient’s survival. Understanding the mechanisms that control UGT2B17 expression in leukemic B cells may provide novel therapeutic approaches in treatment-naïve CLL patients as well as in patients resistant to anti-leukemic treatments.

## Methods

### Cell lines

The B-cell neoplastic cell lines MEC1 and JVM2 were acquired from DSMZ (Braunschweig, Germany) and ATCC (Manassas, VA), respectively. GM12865 and GM12891 are lymphoblastoïd cell lines from normal B-cells of the NIGMS Human Genetic Cell Repository. They were obtained from the Coriell Institute for medical Research (Camden, NJ, USA). Cell lines were cultured in RPMI-1640 supplemented with 10% of FBS, 1% sodium-pyruvate, 1% L-glutamine and 1% penicillin/streptomycin at 37 °C and 5% CO_2_. All culture reagents were from Wisent, St-Bruno, QC, Canada. Cell models were not passaged for more than 2 months and were regularly checked for mycoplasms.

### RNA extraction, and reverse-transcription quantitative real-time polymerase chain reaction (RT-qPCR)

RNA was extracted using RNeasy® Plus Mini Kit (Qiagen, Germany). cDNA was produced by reverse-transcription using SuperScript™ IV Reverse Transcriptase (Invitrogen™, Massachusetts) according to manufacturer’s instructions. qPCR were performed in triplicate using SYBR™ Green PCR Master Mix (Thermofisher Scientific, Waltham, MA, USA) using 10 ng of cDNA. Primers are listed in Table S1, Supplementary file 1.

### Prediction of transcription factor (TF) binding sites

JASPAR: Potential TF binding sites on the UGT2B17 P2 and P3 promoters have been identified using a high-quality TF binding profile database JASPAR2020 ( [22]; http://jaspar.genereg.net/) accessed in September 2020. The search was conducted with the *Homo sapiens* Core transcription factor database using the default relative profile score threshold (80%).

### Reporter luciferase gene assays

pGL3 constructs containing the promoter P2 and P3 sequences have been previously described [2, 13]. Deletions and mutagenesis of TF binding sequences were achieved using the Q5 Site-Directed Mutagenesis kit (New England Biolabs Ltd., Whitby, ON, Canada). The sequence of mutagenesis oligonucleotides is provided in Table S2, Supplementary file 1. The sequence of each construct was verified by Sanger sequencing. Cells were co-transfected with 9.5 µg pGL3 constructs and 0.5 µg pRL-null basic renilla (Promega, Madison, WI, USA) using the Neon Transfection System (ThermoFisher Scientific). Cells were then harvested, lysed and assessed for luciferase activity using the dual-luciferase reporter assay kit (Promega), as per manufacturer’s instructions. Luciferase activity was calculated as the ratio of firefly luciferase to renilla activity, relative to the pGL3 control for deletion constructs and relative to the relevant unmutated pGL3 construct for TF-binding sequence mutations. Assays were replicated four times in triplicates.

### Electrophoretic mobility shift assay (EMSA)

Nuclear extracts from 16 × 10^6^ MEC1 and JVM2 cells were prepared using NE-PER™ Nuclear and Cytoplasmic Extraction Reagents (ThermoFisher scientific) according to the manufacturer’s instructions. Nuclear protein extracts (6 µg) were mixed with biotin-labeled probes (4 pmol/reaction; IDT Technologies (Coralville, Iowa, USA)) and poly(dI.dC) (1 µg) in binding buffer (5 mM Tris, 25 mM KCl, 5 mM MgCl_2_, 0.5 mM DTT; pH 7.5) and incubated at room temperature for 20 min. For competition binding assays, 200-fold molar excess of unlabeled and mutated oligonucleotides were added to the reaction. The sequences of the probes are listed in Table S3, Supplementary file 1. For supershift assays, antibodies listed in Table S4, Supplementary file 1 were included in the binding assay. Following incubation, samples were separated on a non-denaturing 6% acrylamide gel in 0.5X Tris-Borate EDTA. Samples were transferred on a nylon membrane and fixed using UV cross-linking for 60 s. Biotin-labeled probes were detected using streptavidin horseradish peroxidase (LightShift™ Chemiluminescent EMSA Kit, ThermoFisher scientific). Expression of each transcription factor was detected by immunoblotting in MEC1 and JVM2 cell lysates as described previously, with antibodies described in Table S4, Supplementary file 1 [18].

### STAT3 and NF-κB pharmacological inhibition

MEC1 and JVM2 cells were plated 16 h prior to treatment with vehicle (DMSO) or inhibitors. For STAT3 pathway inhibition, each cell line was treated with 10 µM of STATTIC (Cayman, MI, USA) for times indicated in the text. For NF-κB pathway inhibition, MEC1 and JVM2 cells were treated with 2.5 or 5 µM of BAY 11-7082, respectively (Cayman) for times indicated in the text. At the time of harvesting, cells were washed once in cold PBS then centrifuged to obtain dry pellets from which RNA and cell homogenates were prepared.

### UGT2B17 functional glucuronidation assays

Cell homogenates were prepared in PBS containing 0.5 mM DTT. Enzyme assays were conducted with cell homogenates (50 µg proteins) in a reaction mixture containing 50 mM Tris-HCl (pH 7.5), 10 mM MgCl_2_, 5 µg/mL pepstatin, 0.5 µg/mL leupeptin, 2 mM UDP-GlcA, 20 µg/mL alamethicin and 25 µM dihydrotestosterone (DHT) purchased from Steraloids (Newport, RI, USA) as a substrate, in a final volume of 100 µL. Enzymatic reactions were incubated for 4 h at 37˚C, then stopped by adding one volume of 100% MeOH. DHT glucuronides (DHT-G) were quantified using a QTRAP 6500 mass spectrometer coupled to liquid chromatograpy (Sciex, Concord, ON, Canada), operated in multiple reactions monitoring mode (MRM) and equipped with a turbo ion-spray source, in conditions previously described [57].

### Analysis of UGT2B17 genomic features and gene expression

Publicly available ATAC-Seq data from the Rendeiro study [16] was visualized in the UCSC browser (https://www.medical-epigenomics.org/papers/rendeiro2016/#browser). ATAC-Seq data from the Ott study [19] was accessed through the NIH data access portal dbGAP accession number phs001704 as part of the authorized project #25,240. Publicly available gene expression RNA-seq data were obtained from 294 CLL cases of the International Cancer Genome Consortium (ICGC) dataset (project code CLLE-ES) [58] and from the European Nucleotide Archive (ENA) under accession code PRJEB30312 for the leukemia and lymphoma LL100 cell panel, comprised of 100 cell lines representing 22 subtypes of human leukemia and lymphoma including T-cell, B-cell and myeloid malignancies [59]. Transcriptional activity of selected transcription factors was determined using DoRothEA regulons (https://saezlab.github.io/dorothea/) [23] on the normal collection of target genes. Shapiro-Wilk normality test and non-parametric Spearman correlations of high confidence target genes (A and B scores) for STAT3, RELA and IRFs according to Garcia-Alonso [23] and with reported relevance to CLL were determined using GraphPad Prism v9.0 (Dotmatics, San Diego, CA, USA). For overall survival analysis, patients were dichotomized at the median level of TF expression. Survival distribution of the patients in the low and high expression groups were compared by a Log-rank Mantel Cox test (LRT) and are shown as Kaplan-Meier survival curves made using GraphPad Prism.

### Electronic supplementary material

Below is the link to the electronic supplementary material.

Supplementary File 1

## Data Availability

Publicly available ATAC-Seq data from the Rendeiro study was visualized in the UCSC browser (https://www.medical-epigenomics.org/papers/rendeiro2016/#browser). ATAC-Seq data from the Ott study is accessible through the NIH data access portal dbGAP (https://www.ncbi.nlm.nih.gov/projects/gap/cgi-bin/study.cgi?study_id=phs001704.v1.p1) upon approval of access request. Publicly available gene expression RNA-seq data were obtained from 294 CLL cases of the International Cancer Genome Consortium (ICGC) dataset (project code CLLE-ES; https://dcc.icgc.org/projects/CLLE-ES) and from the European Nucleotide Archive (ENA) under accession code PRJEB30312 for the leukemia and lymphoma LL100 cell panel (https://www.ebi.ac.uk/ena/browser/view/PRJEB30312).
