# Supplementary material for: Non-canonical transcriptional regulation of the poor prognostic factor UGT2B17 in chronic lymphocytic leukemic and normal B cells
Source: BMC Cancer. 2024 Apr 2;24:410. doi: 10.1186/s12885-024-12143-7 (PMC10985967; doi:10.1186/s12885-024-12143-7)
Supplement: Supplementary file 1 [file 12885_2024_12143_MOESM1_ESM.pdf]

Non-canonical transcriptional regulation of the poor prognostic factor UGT2B17 in chronic lymphocytic leukemic and normal B cells

Michèle Rouleau[1,2], Lyne Villeneuve[1,2], Eric P. Allain[3], Jules McCabe-Leroux[1,2], Sophie Tremblay[1,2], Flora Nguyen Van Long[1,2], Ashwini Uchil[1,2], Charles Joly-Beauparlant[2,4], Arnaud Droit[2,4], and Chantal Guillemette[1,2,5]

Additional file 1:

Supplementary Table 1: qPCR primers for UGT2B17 variants
Supplementary Table 2: Mutagenesis oligonucleotides
Supplementary Table 3: EMSA oligonucleotides
Supplementary Table 4: EMSA antibodies
Supplementary Data 1: Sequence of the *UGT2B17* gene locus
Supplementary Figure 1: ATAC-seq signals at the *UGT2B17* gene locus in CLL cases.
Supplementary Figure 2: ATAC-seq and epigenetic marks at the *UGT2B17* gene locus in CLL cases.
Supplementary Figure 3: Screening of active cis-regulatory regions in the P3 promoter of *UGT2B17* alt. transcripts by luciferase assays and mutagenesis.
Supplementary Figure 4: Spearman correlations between expression of NF-κB, STAT3 and IRF target genes and *UGT2B17* in the LL100 collection
Supplementary Figure 5: Full blot images of immunoblots presented in Figure 3A
Supplementary Figure 6: Full blot images of EMSA blots presented in Figure 3B
Supplementary Figure 7: Full blot images of replicate EMSA blots related to Figure 3B

Supplementary Table 1: qPCR primers for *UGT2B17* variants

Oligo name	Sequence 5'->3'
UGT2B17_F	TGACTTTTGGTTTCAAGC
UGT2B17_R	TTCCATTTCCTTAGGCAA
UGT2B17_n2_F	TGGCACTTGGAGTCTGGACA
UGT2B17_n2_R	ACACCAGCACCTTTCCACAA
UGT2B17_n4_F	TCTTCAAGGGTTCATGTCTCCA
UGT2B17_n4_R	ACACCAGCACCTTTCCACAA
36B4_F	CCCATGTGAAGTCACTGTGC
36B4_R	GGTTGTAGATGCTGCCATTG

Supplementary Table 2: Mutagenesis oligonucleotides

**Oligo name**	**Sequence 5'->3'**
Deletions	
pGL3_P3_8734_F	CATCGGCAATAATTGCTTTAACC
pGL3_P3_8734_R	ACGCGTAAGAGCTCGGTA
pGL3_P3_8252_F	GAAGCCTAACAGTAGGCG
pGL3_P3_8252_R	ACGCGTAAGAGCTCGGTA
pGL3_P3_7949_F	GCATGCAGCCCCTGTCAC
pGL3_P3_7949_R	ACGCGTAAGAGCTCGGTAC
Point mutations	
pGL3_P3_8734_STAT3mut_F	GCTGTTTTTCCTTTGGAGGATGGGGAG
pGL3_P3_8734_STAT3mut_R	CCTCCAGACTGCCTCATA
pGL3_P3_8734_IRF1mut_F	CAAGGAAGTATTCCCAAAGACAGGCAG
pGL3_P3_8734_IRF1mut_R	TTTTTTCTTAAACATGTACTGAG
pGL3_P2_RELA_mut_F	GTTTCTGCCCTGTTCTGTTTCTC
pGL3_P2_RELA_mut_R	TCAAACAGTGTTCTTCTGTACAATG
pGL3_P3_8734_NFKB1-1_F	ATGGAGGATGAAACGTTTTCCATATCAAAGG
pGL3_P3_8734_NFKB1-1_R	TCCAAAAAACAGCCCTCC
pGL3_P3_8734_NFKB1-2_F	TCATGCAGATAAAAACCTGTTAACTGGG
pGL3_P3_8734_NFKB1-2_R	CCTCTCTTGGTTTCTCCTG
pGL3_P3_8734_STAT3-2_F	TTTTTGGTTCGCCCTTCGGGATCAAATGGC
pGL3_P3_8734_STAT3-2_R	GGCAGAGGTGATAATAGC
pGL3_P3_8734_PAX5_F	ACCAAGAGAGAAACTGCAGATTTTCCC
pGL3_P3_8734_PAX5_R	TTCTCCTGTCCCAAAATC
pGL3_P3_8252_p53_F	CTGCTGCTGGCCTAAACTTAGTAG
pGL3_P3_8252_p53_R	GGGCCAATAGTTTCAGTCCTGGCG
pGL3_P3_8252_CEBP_F	AACATGACTCCCTGCCCTGTTCTG
pGL3_P3_8252_CEBP_R	TGTATATTCTATACATGTCTGGAATCTATGAATAAC

Supplementary Table 3: EMSA oligonucleotides

Oligo name	Sequence 5'->3'	5'end modification
IRF1-F-5'biotin	AAAACAAGGAAGTAAAACCAAAGACAG	biotin
IRF1-F-unlabeled	AAAACAAGGAAGTAAAACCAAAGACAG	-
IRF1-R-unlabeled	CTGTCTTTGGTTTTACTTCCTTGTTTT	-
IRF1mut-F-5'biotin	AAAACAAGGAAGTATTCCCAAAGACAG	biotin
SPI1/IRF1 mut-F-unlabeled	AAAACAAGTCCGTAAAACCAAAGACAG	-
SPI1/IRF1 mut-R-unlabeled	CTGTCTTTGGTTTTACGGACTTGTTTT	-
STAT3-F-5'biotin	GGGCTGTTTTTTGGAATGGAGGATG	biotin
STAT3-F-unlabeled	GGGCTGTTTTTTGGAATGGAGGATG	-
STAT3-R-unlabeled	CATCCTCCATTCCAAAAAACAGCCC	-
STAT3mut-F-5'biotin	GGGCTGTTTTTTCCTTTGGAGGATG	biotin
STAT3mut-F-unlabeled	GGGCTGTTTTTTCCTTTGGAGGATG	-
STAT3mut-R-unlabeled	CATCCTCCAAAGGAAAAAACAGCCC	-
RELA-F-5'biotin	GAACACTGTAAAACCCCCTGCCCTG	biotin
RELA-F-unlabeled	GAACACTGTAAAACCCCCTGCCCTG	-
RELA-R-unlabeled	CAGGGCAGGGGGTTTTACAGTGTTC	-
RELAmut-F-5'biotin	GAACACTGTTTGAGTTTCTGCCCTG	biotin
RELAmut-F-unlabeled	GAACACTGTTTGAGTTTCTGCCCTG	-
RELAmut-R-unlabeled	CAGGGCAGAAACTCAAACAGTGTTC	-

Supplementary Table 4: EMSA antibodies

Target	Antibody	Quantity in assay (µg)
IRF1	IRF1 E4 X sc-514544 (Santa Cruz, California)	1 µg
STAT3	STAT3 polyclonal antibody (ProteinTech, Illinois)	0.5 µg
NF-kB (p65)	NF-kB p65 D14E12 XP® (Cell Signaling Technologies, Massachusetts)	0.5 µg

Supplementary Data 1

***UGT2B17* gene locus (**GRCh37/hg19 chr4:69,448,292-69,433,471)
Note that *UGT2B17* gene locus is located on the reverse strand.

Genomic sequence up to exon 1 is provided.
Exon 1d
Exon 1c
Exon 1b
Exon 1
The sequence is annotated to indicate positions of chromatin features shown in Figure 1F.
Coordinates are given relative to the translational start site (+1-ATG codon)
Red labeled nucleotides indicate sequence discrepancies between GRCh37/hg19 and material derived from human cancer cells used in this study.

TCAGGTCACTGGAGTTGTGTACCTAGGTGGATTTTGGCTGCCTCTGCTGAGTCATGAGGTCGTCAGGGAAGTGTGGGA
AAGCCAGCAGTCTCAGTTCTAACCCACCTCCCATGCACACTGAAGGGCCACTCCCACTTCCACCGTGGCCCCTGCAAT
AGTCCCGAGTCTTTTTCAAGGAGGAGGGAGAGTTGGACTTGAATATTTGCCTGAGATTTTCCACCTCCCAGCTGCAAA
GAAAAGGGCTTTAGTTCTTCCCCTGCCTGTGAAGTCTGCAAGCTAGATTCATGCCCTCCCCCAATTTCTGGCCATGAG
GCTTCTTGCCTGTTCAAATTGTTACAAAGTTCAGCTAGAGAAGTCCCTCTTACTGTGGAGTTTTACCCCTGTAATGTT
CTTGTATCGGTTCAAACCCTGAGAGCATGCCAACAGAAACCACGAGGTGGTGTGGAGCAACATGCTGTTTTAATGAGC
ACCTGGGTGCAGGTGGGCTAGGGCCTAAAGGGGCGTCAGCCCCAAGTGAGGATGGGACAGGGGTTTTGTAGTCCTCTG
TAAACAGTAAGTGTCCCAGTCTGATGTGACTGCTATGTAGTACCCAGACGGTCTCTTTCTTGATCTTCAGGGGTATGT
GTCTTCCAGCCAGGGTAGGTGTCTTCCAGTTGGCTCTCTTCCTGCTTCTGCTATCTTTCTGATGCAAGCTGTTGACAC
AAATGGCTTTGCATCTTGGAACTGGGCCTGAGAGGAGGAGTTACTCATCCCTTCAAGCTTTCAGGCCCCAGGGAGAAT
CTTCCTGTTTATTTGGTTATAGAATAAAGGGAAAAGGGGTGACTTTCTCAGTAACTACTTCAGGCATGACACAGGGAT
TGGAGTGGGCACCTTGGAAAAAAAAAACTTAATTTTTGGGGTATTCTTGAGAGACAGGTTGGTATCCACCATGTCGCT
GTAGCAGGAGCACCATCTGTATTGTCTGGAAACATGTCTGTGCCTGCAAGTCAGTTATGTTGAGAGGACCAGGAGGTG
TCGACAGGGAGAGGCATGGCTCATTTGCTGCTTCACAAATGAAAGACCATGTCTGGATTAAGGAGGGGTGGTAATTTC
TGAGTGGCTCAGAGTCTGGTAAGGGTGGAGGCCCCAAGACAAAATTTTGGTCATACATGATTTCAAAGGGACTATAAA
AAGAGTGTGCTTTTGGTGTTGTGCAAAGTCTTATGAGGGCGAAAGGGAGATTTTTTTTGTCCACGACTGTTGGGTTTC
TACAGCCAGCTTGGTGAGTTGGGCTTTAAAGACAGAGTTAATTTTTCTAACTTCGCCTGAAGATTGAGGCCTGTAGGG
TGTGTGGAGAGCTTACTTTATTCTTAAGGATGTAGAGATGCCTCGGGTAATTTGGCTGATGAAGGCAGGACTGTTATC
AGACTGGATGGATGTTGGGAGTCCAAAACGGGGAATTGCAGGTATGCATGATGAGTTTGTGCGATGACAGTTGCACCT
TCTGAAGTTGTTGGGAATTCTTCTACCTACCCAGAGAAAGTACAGACAAGGACTAGAAGACTTGTCTTATGACAGGAG
TATTGTTTGCAGTTAGGAAACCCCGTTCTTTCCAGATGGATGAGTGTGAGTGCACTACGTGGAGCGCATAATGAGAAT
TTGAATAAATTGATCTGTTTTCCGGATGCTAGAGTGAGAGCTGGAGTGAGGGCATTGAATTCAGCTTTTTGGGAGGTG
GTGCCTAAGAGGAGCGGATTGGCTTCAATAGTGTTGGGGGGTGACACTATAGCATAGCCCCCATGTCAGTGTCCTTGA
TGTAGGAAGGAGCTGCCATGTACAAACCAAATAAAGGAGGCATCTGGAAGGGATTGGTCTGTTAGGTATGGAAAAGGT
ATAAGAAAGGTTTGAACAGTATTCACACAGAAGAGTGTAGAGTCTTGGGCAGTTGTAGCTTCAGGTAAGAGCATGGAC
AGGTTTAGATGGGAGGTGGTTAGCATGGTGATGTGGGGAGTTTCTATGAATAGAGCATACAGCTGGAGGAGCCGTGGG
GCAGAGAAAAGACTTAGTACACTGCGGTGAGCTAACATGTCTTTGATGTTATGGGTTGAATAAACTGTTAGGTTGGCA
TGAAGAGATAGTATTAGGCTTTTAAGAGTGAGGAGAGCAGCTGCCACCAATGCTCAGGGGCAGGCAGGCCATCCAAGA
ACTGTGGTCTCAAGCTCTTTAGGGAGGTAGGCAACAACCTGGCCGGGTGGGCCCCTTAGACTGGGTTAGAACACCTAG
TGCAACTCCACGCCATTCATCAGTATAAAGGGAGAAAGGTTTGGTGAAGTCTGGGAGAGTGAGGATGGGGGCTGAGAT
GAGAGACTTTTGGAGTAGATGGAAAGGTTGGGTAATAGGCTGTGCAGGTTTTAAAGGCTCATAGAGAGGGACTTTAAC
AGCTTCGTATAATTTTTTGGCAAGTAAAGCGAAGGAGGAAACCCAGAGCCTAAAATATCCCGCTAATCCTGGAAAAGA
GAGAATTTCTTGCTTAGTTTGTGGAGGCGGGAGGGACTGAAGGAGGGAATATGTGGTCTGTTGTGAGCCCTTGCGTTC
AGGAGTGAGAGCTAGGCCTAGATAGGTGACTGGGGGGTGCATATTTGGGCTTTCTTAGGGGAGACCTGATACCCCTGT
TCTGCCAAGAGGTTTAAAAGAGAGATAGTATGTATGGGTGTTGCAGTCTCTTTGAGAGGGGCTACACAGGAGCAGATT
ATTAACATATTAAAGAAGAGAGGATGATTTTAGGGATAAAGTACAGAAGTTACTAGCAAGGGCCTGTCCAAAAAGGTG
GGGGCTGTCTCTAAAACCTTGAGGTAGTACACACGAGGTGAGCTGACCTGAAAGGTGGGTGTCGGGGTTCTCCCAGGT
AAAGGCAAAGAGATTTTGGGAATCAGGGTGTAAAGGAATTGTGAAAAAGCATCATTTAGGTTTAGAACAGAAAAATGG
GTGGTATTCGAGGGAAGTGAGGAAAGTAAAGTATATGGGTTAGAAACTACTGGACACACTGGGAGTACAGCTTGGTTA
ATGAGCCTGAGGTCCTGGACTAAGTGATAAGTTCCATCTGGCTTTTTAACAGGTAGAACTGATGTGTTAAAAGGAGAG
TTTGTTGGGCAGAGTAGGTGACTGGTGAGGAGGAGAGAAATGATAGGCTTTAGGCCTATGAGAGCTGCTTGGGGGATG

Commentaire [MR1]: -13709: First nucleotide of HERVH48-int

Commentaire [MR2]: -13562: First nucleotide of exon 1d

Commentaire [MR3]: -13454: Last nucleotide of exon 1d

AGATACTGCTTCTGTGATAGGAACGGGGTGGGCTCTTTAAGGGTGATGTGGATGGGGGTGTGGTGCTCTGTGACCGAG
GGTGTGGAAGTATCCCAAACAGTGGGGTTAACTACAGGTGCGGGATAAGGAAAAGTTGCATGCTTCAGGGTGGGAGGT
CGGAGGAGTAGAAGAAAGTTAGAAGCCTGTTAGAAGCCTTGGAGGGGTCTGGGTTGATGTGTTGGGTACTATGGGGAA
TGTGGAAGTGGAGAGTAGTGTGGAGTTTTGAAAGAATATCTCTGCCTAGGAGCAGAGCTGGGCATGAAGGCAGGACTA
AGAAAGAGTGACTGAAGGAAAAGGTGTGCAGGGAGCAGAAAAGTGGAGGGGGGTCTTGGGGTTTGGAGACTTGTCCAT
TGATTCCCACAACAGAGACTTGGGAGGACTGGGTGGGTCCTGAAAATTTAGGTAAAGCAGAGTAGGTTGCCCCAATAT
TAATTAAAAAAAAAATACTGGCCTATCTGCCAACATCAAGGTTATCCTTGGCTCAGATGAAGCAATGGTAGTTGCTGG
GGCATCTGTTCCAGGGCACTGTCAGTCTTCAGCGGCAAGGCTGATGAGATCCTAGTAGGAGGTTTTGGCCGGCTGGGG
CAGGGATGGGGGCGGTCCTTGCTGGGGCACCTCACAGTCTGACTTCCAGTGGGGTCCTCCGCAGAGAGGGCACAGCCT
GGTGGGCTTACCTGGGTTTGGGCATTGCCTGGACCAGTGGCCTTCATTGCCACACTTGAAACAGGTGCCAGGGGGAGG
TGGATTGCTAGGAGGCATCTGTGTGGAGCTGCAGCCTGTGGGCCTGCAGGGCCCGTGATGGTGGAGGCAAGCATTTGA
AACTCTGTCTTTTTTTTTTTTCTTTTACTTTCCTCGTTATAACTGTTAAAGACTTTGAAGACTAAATTAAGAAGGTGT
CATTGTGGGGTTTCAGGGCCATTGTCAAGCTTCTGAATCTTGCACCAAATATCAGGGGTAGATTGGATGAGGAACCAA
AGGTTTAAAATAGTGATTCCTTCTGGGTTGGCTGGGTCTAGGTTGGTATATTTTCTCATGGCTTCAGTTAAACAAGAG
AGAAAAAAAGGCTGGGTTTCTGTCAGGACCTTGGGTGATTTCTGAAAGGTTTTTATAGTTTACCACTTTATGGCACCC
TTTTTGAGTCCTGCAAGGAGATACGCAATCATGTGGTCTTGATGGCAGTGTCCAGAGGCCCTGTCTTGATAATCCTGG
TGGGGCTCCTGGTTGGGGACTGCCTCCGTGCCAGTAGGCTGGGCAGGAGCTTGATGATGAATTGTATTAGCATGCTCC
TGAACCAGGGTCCAAATATGGTCCCTGTCTTCTGGGGTGAGGGTGGAAGAGAGGATAAGTAGAAGGTGAGGGTGGAAG
ACAGGATAAGTAGAGGTCATACCAGGTTAGTGCATAAGACTGGGTAAGGTACTGAAACTCCCTAATATAAGAGGTAGG
GTCTTCTGGAAATGAACTGAGTCTTGTTAGTTTGAGAGATCAGTGAAGGAGAAGGGAACATGAACTCTAACAATACCT
TCAGTTCCTGCTACTTCCTGAAGGGGGCATTCTAGCACCCGCACTGAAGTATGGGTGCAGCGTGGGCCAAAGATGGTG
CCCAAGCTGGTATGGACAGGAGAGAAGAAAGAAGCCAGAAGGGGTTCCTGCTGAGGGTTTGAAGGGGGAAGGCGGGTT
GAGTTGACAGGCAGTGGAGGATACATAGGGGTGTAAGGTGGCTTGATGGGTTTACAGGCTTCAGGAGAGGGCAGTGAG
GAAGGAGACTGAGTACAGGCAATGCTAGAGTTTTCCTGAGGAGAGGACGGTGTAGAAAAAGAAGTGGGTACTTTTGGA
GGTGATGCCGGCTGGGAAGATGGTGGCTGGGAAGATGGTGACTGCGAAGACAATGAGAAAGCTTGGGGAGTTATAGAA
GATGGTTGAGACGTGGGGGTAGGGGCTGGGAGGGGTGGACAGCAGTCTGCCGGATCCAACAAGGAAAAAGAGGTAGGG
TCAGGAGGAGAAAGGTTATGAGGGTGGTGAGAATGAAGGATTTGAACAGGTAAGCAAGAATTGCAGAAGTCAGGTTGT
GATCTGAGTGCAAAAAGGCCTGGAACAAGGAATTTCTCCCCATTTTTCCAGTCATCGGCAATAATTGCTTTAACCAGT
TAAAATTGTAAAGTCAAGTGTTTCATTTGCTGGCCATTTGGACCCGTTATCTAATTCATACTGCGGCCAGGTTTTATT
GTATAAAAAAGACAAGGCGCTTAGGGTGGGTATCTTGCCTGAGGCCTAAGGTTTGCAGATTTTTTATGAGGCAGTCTG
GAGGGCTGTTTTTTGGAATGGAGGATGGGGAGTTTTCCATATCAAAGGGTAGGCTCTGGAGAACAGGGAAAAAGAAGA
CTGTCCTGGACAGCTGGAGGAAGACAATAAAAGGAGCTATTATCACCTCTGCCTTTTTGGTTCCTGGAACGGGATCAA
ATGGCTTAGAGGCATCCCCCTAAGACCAGATGATCAGGGAGTGCCTGGCACATACTGGAGCCTTCTTGGATCAGTGTT
GGATTTTGGGACAGGAGAAACCAAGAGAGGTCATGCAGATTTTCCCCTGTTAACTGGGCTCCCAGGGAAGCCTAACAG
TAGGCGAGATCAGTGACCAATGTGCATGTGAAGGAAGAGAAAGACCTTCTCATATTATTTTATATTGTTTTTCACTCA
GTACATGTTTAAGAAAAAA~~CAA~~CAAGGAAGTAAAACCAAAGACAGGCAGCCCAAACGCCAGGACTGAAACTATACCTG
GGCCTGCCTGGCCTAAACTTAGTAGTTAAAACTCAACTCATAACTTAGAAACCAATGTTATTCATAGATTCCAGACAT
GTATAGAAGAACACTGTGAAACTCCCTGCCCTGTTCTGTTTCTCTCTGACCACCAGTGCATGCAGCCCCTGTCACATA
CCCACTGCTTGCTCAAATCAATCACGACCCTTTCATGTGAAATCTTTAGTGTAGTGAGCCCTTAAAAAGGACAGAAAT
TGTGCACTCGGGGAGCTCAGATTTTAAGGCAGTATCTTGCCAATGTTCCCAGCTGAATAAAGCCCTTCCTTCTACAAC
TCGATGTCTGAGAGGTTTTGTCTGTGGCTCGTCCTGCTACATTTCTTGGCTCCCTGACCCAGAAGCAAGGTGACTGAC
GGAAGGCTGAGGCAGCCCCTTAGGTGGCTTAAGCCTGCCCTGTGGAGCATCCCTGTGGGGTACTCCGGGCAGCCTGAG
TGACGAGATCCAAAGAGCGCTCCTGGGTACGAAATTGCCCCAGTGGAACACCTCACCAGATCAACACGTAGCAGGCCC
CCGTGGAGGATTAACACATTGGCTGAACACCGGGAAGGAACTGGCACTTGGAGTCTGGACATCTGGAACTTGGTAAGA
CTAGTCTTTTGAACTTGTCCCACTCCACCTGAGTAGAAGCATGGCCTGGTCACCCACGGTGTGCCTGTGTTGGCACCT
TTGTTCTGGTTTTGACTTGGCTAGACTTGGTAGGACTAGTCTTTTGAACTTGCCCCACTCCACCTGAATGGAAGTGTA
GCATAGCCTGATCACCCACGGTGTGCCTGTATTGGCACTTTTGATCTGGTTTTGACTTGACTTGAATTGCTGGATACT
TTGGTTTTGGTTTTTGACCTGGCTTGGATTTCTGGATACTCTGATTTTGGTTTTGATTTTGGTTTGGTGTAAACTGAA
ATGTTTGTGTGTGCCCTTTTACCCGTTCTTTGTTTTGTGGTGTGTGTGTGCTGTGAGCATGGTGTTTTGTCTCAAAAA
AACATAGATCAGGCACAAAGTAAGCCCACTGCACTGGGAACTATGATAATGAATTTCAAGAAAGGATTTAATAGAGAC
TATGGAGTCACTATGACTCCAGGAAAACTTAGAACTTTGTGTGAAATAGATTGGCCAGCATTAGAGGTAGGTTAGCCA
TCAGAAGGAAGCCTAGATAGGTCTTTTTAGTTTCTAAGGTATGGCACAAGGTAACCTGTAAGCCAAGGCACCCAGACC
AGGTTCTGTACATAGACAGTTACAGCTGGTTTTAGACCCCTTCCCCCCACCCCCCCTCACAATAGTTAAGAGAACAGC
AGCATAAGCAGCTGGCAGAGGCAAGGAAAGACCAGCAGAGAAAAAAAAAAAAAAGGCCATCTATACCAATTCTAAGTA
AATTTAGACTAAACAAGGTCTTATTAATAGCAAAGGATAATTGAAATTTCAAACTTACAAGGTTTTCAACAAAAGCGA
AGTTTGCTAAAAGTTGACAGTGTAACACATATTATGGTAACTTCTAATCTTGTGGCCTTAGACAGTCTAGTCCAAAGA
CATAAAGAAAGTTCACTTTTTAAAAAAAAGGAATGGTTATCTTCAAAAAAAAAAGGGGGGGGAGGAGGTAAAATTTAT

Commentaire [MR4]: -9471 to -9039: CTCF bound region

Commentaire [MR5]: -8544 to -8534: STAT3 binding site

Commentaire [MR6]: -8374: First nucleotide of ATAC-seq peak (Rendeiro et al)

Commentaire [MR7]: -8212: First nucleotide of Harlequin-int

Commentaire [MR8]: -8209: First nucleotide of POL2A bound region at exon 1c

Commentaire [MR9]: -8153: First nucleotide of DNAse1 hypersensitive region #1

Commentaire [MR10]: -8137 to -8126: IRF1 binding site

Commentaire [MR11]: -7949: First nucleotide of exon 1c according to RNA-seq of MEC1 cells (Allain et al, Front. Oncol 2019)

Commentaire [MR12]: -7879: Last nucleotide of DNAse hypersensitive region #1.

Commentaire [MR13]: -7838: First nucleotide of exon 1c in NM_001077.4

Commentaire [MR14]: -7466: Last nucleotide of exon 1c

Commentaire [MR15]: -7448: First nuclotide of DNAse hypersensitive region #2

Commentaire [MR16]: -7364: Last nucleotide of Harlequin-int

Commentaire [MR17]: -7358: First nucleotide of Harlequin-int

Commentaire [MR18]: -7284: Last nucleotide of DNAse hypersensitive region #2

Commentaire [MR19]: -7179: Last nucleotide of POL2A bound region at exon 1c

Commentaire [MR20]: -7146: Last nucleotide of ATAC-seq peak (Rendeiro et al, Nat Comm 2016)

GTAAAAAGAGTGTTATATGGTAAATTCTTGTCCTGAAATAAATTAACTTGTTGTTTAAAGAGAAGAAAAAAATGTTTG
TAATAAGTCAGAAAGTTGAGACATGTTGAAAAATTGTTGGCGAAAGTCGTGAAAAAAAGTTATAAAAAATTTTATGCA
AAAAATGTTGCATAATTTAAAAGTAATAAGGCCTCCTGAGTGCTATTGAAGAAACAGTTTATGTGCAGGTGTATAAAA
AAGTAAAATATAACTTTAATGAGAAGATTATAAGGAGGCATAAGAATGTGGATTTTTACCTACATTAAAAAGTCAAAA
AAGAATTATTGTTTGAAAAGTTCAAGCAAGTTTTAAAATGTTAATTTAAAGAAAATTCTGTGTGTAAACTTATTAGCT
AAAGTTAAAAAAGTATTATCCAGTTTTTCTGTGAACTAGACATGAAAGTAAAAATGCAACAGGTTTTTTCCTTAAAGC
ATCAACCTGCTCTTTAACAAAAATTGTAAAAGGTTAAACAGAGTCTATAAAATCTTACCTTATGGTCAACTATGAAAA
ATTGGATAAATATGTCTACAAGGTTTTATTAAAATTAAGTTTAATATTAATAACACACTAATATAAAAGTAAAATTTA
GCTTATCTGGTATAAAAATCATACAAGAAACATTAAATATAAAATGATGTTTAGCTTTCTTTGGTCTAAAAACTAATA
AAAATTAGTGCTAATGGAAATATTCATTTTACTAGAGGATCATAAAAGTTAAAGACTTAAAACAAACTTTAGCAATTA
AGACAGTATATCAAGCTACAAATGCCTGGTTGAAATGGATCAAATATTCCAACTACACAATAAACAAAAGCAATTGTT
ATGCTTGTGCACATGGCAGGCCAGAGGCCCTAATTGTCCCCTTCCCACTACGGTGGTCCTCCAGTTCACCAGGTGTGG
GCCTGATGGTAGCTCTTTTCCAGGATTCTACAGCCTGGAGTAATAAGTCATGCCAAGCTCTCTCTGCTATATCCTGAA
GTCCCTGCCAGTCAGCCCCCAAGGGCCATCCAGCTTCTGTCTCTCAACACTAAGTTCACTTCCTGTCTCTCACGGCAG
GGAGGAAACTTAGCATTCCTTGGAGACCTAAAAGGATGCAGTGAGCTTAAGAATTTTCAAGAGCTTATCAATCAATCA
GCCCTTGTTCATCCCTGAGCAGATGTGTGGTAGTATTGTGGTGGACCTTTACTAGGCACTCTGCCAAATAACTAGAGT
GGCATTTGTGCCTTAGTCCATTGGGCTATCCCTTTCACCCTGGCATTTCATCAACCAGAGGAAGGAAAAAATAATAAT
AACACATCGTAAAGTGAGAGGAGCCCCTTATAAGTCTTTCAACTCTCACATCTATTTAGATGCAATCGAAGCCCTGCA
AGGAATACCAGATCAATTTAAAGCTTGAAATCAAATAGTTACAAGATTTAAGTCAATATTTTAGTAGATAACAGTCAA
TAAAAATGTAGATTAGATAAACTACATCTATTACAACCAACAGCAAAGAGCTTTTCATGAGTTAAAAAAAAAAAAAAA
ACTCATGTCGGCCCCAGCCCTGAGGCTACCTGACCTGACAAAACTCTTCACACTCTATGTGTCAGAAAGAGAAAAAAA
TGGCAGTTGGAGTTTTAACCCAGACTGGAGGTTCCTGGCCAAGGCCAATGGCCTGTCTCTCAAAACAACTAGACGGGG
TTTCCAAAGGCTGGCCCCCATGTCCAAGGGCCCTGGCAGCAACAGCCCTGTTAACACAAGAAGCAAATAAGCTAACTC
TTAGGAAAAACCTAAACATAAAGTCTCCCCATGCTGTGGTAATTTTAATAAATACCAAAGGACATCATTAGCTAATAA
ATGATAGACTAACTAGATACCAAAGCTTGCATTGTGAAAATCCCCACATAACCATTGAAGTTTGCAACACCCTAAGCT
CACTATCTTACTCCTATTATCAGAGAGCCCAGTTAAACATAACTGTACATCAGTAGACTGGGAGCTGTACGTGGATGG
GAGCAGCTTCACCAATCCCTGCAAAATGACTCTGAAGAAGACAACAAACACTGCTCCAGTCACACCCAGAAGCTGACT
GGTCCACGCATGGCTGAAGCATGAGGAGACTCATCGCAGGACTCATTTTCCTCAGAATTTGGACTTGTACAGGAAGGA
CTTCAACTGACCTTCCTCAGACTAAGAACTGTTTCCAGTATATACATCAAGTCACTGAGGTGGAACAAAAGATTGCTA
CAGTCCTATTACTTTATAGTTATTATAAAAGAGCTAATTACTTTATGGTTATTATAAAAGAGCTAATTGCCTTCCATG
ATCACGCCCTCATAATCGTCTTTCTTATCAGCTTCCTGGTTCTATATGCCCTCTTTCTAACACTCACAACAAAACTGA
CTAACACTAATATCACGGACGCCCGAGGACTCTAAAAAAATTAAATAGCAACAAAAACAACAACAAATTGTTTGTATA
ATGCTATTCTATCCAAGGTATGTAGCCCAGGAAATAACCAACCTGATGTGTGTTATGAACAATTTTAAGCCTCCCATG
ATCACAGTTTTTAAAATAAAATTAAGGACTGCTCCTTTTCTATGTGACACAAGTAAGGTAATAGGTAGAACCGAAAAA
AACAGAGCTCCTCAAAATGTAACCTTAAAATTTGACACTTGTGCCGCTATTAATAGTAAGCAGCATGAAACAGGATGT
GATTCTCTAAATTAAAAAAGAAAGTTACACAGTAAAAAAAATAAGTATATCTGTCAAAAATCATATTTATGTGAGATG
TGTCAATACTGGTCTCGTGTCATTCAGACTACTTAAAAGGAAAATAAAAAAGATCGTGTTTGACTCCAAAAAGGAAAA
CTCAGCCCCTCTTGCATGAATAGGAGCTACAACCTTTTAGAATTGATAATCACAAACCCCTCAGACCCAAAGTAAAAT
AAAAGAAAAATATATATCATTAGACATGGATAAAAAGGGACTAGATCCTAATGTAAGCATCCTAATAAAAGGAGAGGT
TCAAAAACGCTCTCCAGAACCAGTGTTTCAGACTTTCTATGATGAACTAAATGTGCCAGTACCTGAGATTCCAGGAAA
AACTAAAAATTTCTTTTTGCAATTAGCCTAACACGTAGCCCAGTCTCTACAAGTCACCTCATATTATGTTTGTGGAGG
AACCATAACAGTAGATCAATGGCCATAGGAAGCCCAAGAATTAGTTCCTACAGACCCATTTCCTGATGAATTCCCAGC
CCAAAAGAATCACCCTGATCATCTCTAGGTTCTAAAAGTCTCAATTATTAGACAGTATTGCATAGCTAAAGAAGGAAA
AGGATTCACTCATCCTGTAAGGCAGCTTAGCTGTCTTAGACAAAAGCTCTATAATAGTACCACAAAAACAGTCACATG
GTGGAGTTCCAATTACACAGAAAGAAATCCATTCAGTAAATTTCCAAAGTTGCAGACTGTTTGGGCCCACCCAGAATT
CCACTGGGACTGGACGGCCCCCACCGGATTATACTGGATATATGTGGACACAAAGCTTATGCTAAGCTGCCTGATCAG
TAGACAGGTAGCTGTGTAATTCGCACCATTAAGCCTTCTTTCTTCTTACTGCCCATAAAAACATGAACTTCTAGGCTT
CCCAGTCTATGCTTCCTGGGAACAAAAAAATGAAGCATAGCCATAGGAAAAGATGATAAATAGCCCCCTGAAAAAAAT
CATACAATACTATGGACCCACCACTTAGGCACAAGATGGCTCATGAGGATATTGGACCCCCATCTACATGCTCAACCG
AATCATACGGTTACAAGCTGTTTTAGAAATTTTTACTAATAAAACCGGTCAAGCCTTGACCGTTCTTGCCCGGCAAGA
GACTCAGATGAGAAATGCAATCTATCAAAATAGACTAGCTCTTGACTACTTGCTAGCAGCTGAAGGAGGAGTTTGTGA
AAAATTTAACCTTACTAATTACTGTCTACACATAGATAATCAGGCAAGTTATTAAAGACATAGTTAAAAATATGACAA
AACTGGCACATGTACCTGTGCAAGTGTAGCACGGATTCAACCCTGAAGCCATGTTTAGAAAGTGGTTCCCAGCACTAG
AAAAATTTAAAACTCTTATAATAGGAGTTACAATAATTATAAAAACCTGCTTACTGCTCCCTTGTTTGCTACCTGTAC
TTCTTCAAATGATAAAAAGCTTCATCGCTACCTTAGTTCAACAAAATGCTTCAGCACAACTGTACTATATGAATCACT
ATCAGTCTATTCCACAAAAAGACATAAGTAAAAAAATGAGTGAGAACTCCCACTAATAAAAAGTGAGAGTCTCAAAAG
GGGGGAATGAAGGAAGAGAAGGACCCTCTCAGATTATTTTATATTGTTTTATGCTCAGTACCTGTTTTAAGAAAAAAC

Commentaire [MR21]: -2011: First nucleotide of ATAC-seq peak near exon 1b (Rendeiro et al, Nat Comm 2016)

AACAAAGAAGTAAAACCAAAGACAGGCAGCCTGGCTCCAGGCCTGAAACCAGGCCTGGGCCTGCCTGACCTAAACCCA
GTAGTTAAAAATCAACTCATAACTTAGAAACCGATGTTATTCAGAGATTCCAGACATTGTACAGAAGAACACTGTAAA
ACCCCCTGCCCTGTTCTGTTTCTCTCTGACCAGCAGTGCATGCAGCACCTGTCACATACCCACTGCTTGCTTAAATCA
ATCACGACCATTTCATGTGAAATCTTTAGTGTTGTGAGCCCTTAAAAAGGACAAAAATTGCGCACTTGAGGAGCTTGG
ATTTTAAGGCAGTAGCTTGCCGATGCTCCCAGCTGAATAAAGCCCTTCCTTCTACAACTTGGTGTCTGAGAAGTTTTG
TCTGTGGCTCATCCTGCTACACATGCACAGAGAGGCAACTGGAGGCTGAGGAGTTTCCTTTGTCTGGTTGCTGTGGCT
TGCTCTCTGGGGTGGAGGGGTAGGTCCACAAGGGACACAGACCTGAGCCCCACCCAGGTTTTGGCACCAGATATAAGG
TTCTTGAATTGGTTTGATCCCTGAGAGCATGCCAACAGACAACACGAGGCAGTGCGGAGCAGCGTGCTGTTTTAATGA
GTGCCTGGGTGCAGGTGGGCTGAGGCCTAAAATGGCATCAGCCCCAAGTGAGGATGGGACAGTGGTTTTATAGTTCTC
TGTAAAGAGTAAGTGTCCCAATCTAACGTGACTGCTATGTAGTATCTGGATGGCCTCTTGATCTTCAAGGGTTCATGT
CTCCAGCCAGGGTAGGTGTCTTCTGGCCGGCTCTCTTCCTGCTTCTGCTATCTTGCTGACACACGCTGCTGATGCAAG
CAGCCTTGCATCTTGGGACTGGGCCTGAGAAGGGAGGAGTTACTCATCCCTTCAAGCTTTCAGGCCCCAGAAGAATCT
TTGAACCCCCTGCTCCGCTGGCCACCCTCCTAAAGGATCACTGTGGTGCCAGGCAGGAATGAGCTGCTTGGGTATCCA
GAGAGCTCCCAGTACCTTTCTGCTACTTCCTCTACCCCTATATTTTGCTTGGCTTGGTTCTCTAATTTGACTCAGCTT
CACATAAAGTCAGGAACTTCTCCTGCAAACAGAACTTCAGCTTCTCCAGTGGGGATGTGTATTCTGGAGAGGGGGATC
ACCCTTTCCCACTTCCATTGTTGGGGCACTCACAGTGTTTGGGATGTCTCCCAGGTCCTGCAGGAGCAGTACGCTTCC
TGCAGAGGGTGTGTGGATCCTCTCAGAATTGCTGGTCTGTCCTTGCAGTTGACCTGCAGCTAAAATTCACAATGCAAG
CCTCTGCATGCTGCTCTGTCTGGAGCTGCAATCTAGTCCTGCATCCCATCTGCCATGATCACTGGAAAACCCTCATTT
ATTTTTTAAAGGGTCCAGAAAATGCTAATCTATAGAGATAGAAATTAGATTAGTGGTTGCCTAGGGTAGGATGGATGC
AAAATTTCAGAGTGGGGGGTTAGAGGCTATTGTATAGAATCTTTTGGAGATAATACTGATTATTGTAGTGAAAGTAAA
ATTCTGTGAATATACTAGGAAACATTGAACTGTACACACTAATTGGTGAGTCATATGGTATATGAATTATGTGTCAAC
AAAGTTTTAGAAGACATTACTTGCACCACGATATTAAAAAATGGCGTTTGAGTTGTATAATTACTTCTTCTCTCTATG
TCAAGGGCACCGAACAGGCAGGAGCCTCTCACCTGCCACTGTTCTTAACAGTATTATAAAATAATTACATAAGACAGG
TTACTTACGTATTCTAGGTCATAAAAATTATTGCTTGACTAGAGTAATTGTAAATATAAAAGAACACCAAACACACTA
AAATAAATATGAGGTCAACTCAAATTTTAGCAGTTATATTTTAACTTGATTGATTTTTCCTCAGATATAAGTATGAGA
AATGACAGAAAGAAACAACAACTGGAAAAGAAGCACTGCATAAGACCAGGATGTCTCTGAAATGGATGTCAGTCTTTC
TGCTGATGCAGCTCAGTTGTTACTTTAGCTCTGGGAGTTGTGGAAAGGTGCTGGTGTGGCCCACAGAATACAGCCATT
GGATAAATATGAAGACAATCCTGGAAGAGCTTGTTCAGAGGGGTCATGAGGTGATTGTGTTGACATCTTCGGCTTCTA
TTCTTGTCAATGCCAGTAAATCATCTGCTATTAAATTAGAAGTTTATCCTACATCTTTAACTAAAAATGATTTGGAAG
ATTTTTTTATGAAAATGTTCGATAGATGGACATATAGTATTTCAAAAAATACATTTTGGTCATATTTTTCACAACTAC
AAGAATTGTGTTGGGAATATTCTGACTATAATATAAAGCTCTGTGAAGATGCAGTTTTGAACAAGAAACTTATGAGAA
AACTACAAGAGTCAAAATTTGATGTCCTTCTGGCAGATGCCGTTAATCCCTGTGGTGAGCTGCTGGCTGAGCTACTTA
ACATACCCTTTCTGTACAGTCTCCGCTTCTCTGTTGGCTACACAGTTGAGAAGAATGGTGGAGGATTTCTGTTCCCTC
CTTCCTATGTACCTGTTGTTATGTCAGAATTAAGTGATCAAATGATTTTCATGGAGAGGATAAAAAATATGATATATA
TGCTTTATTTTGACTTTTGGTTTCAAGCATATGATCTGAAGAAGTGGGACCAGTTTTATAGTGAAGTTCTAGGTAAGT
CG

Commentaire [MR22]: -1853 to -1839: NF-kB binding site (Encode ChIP-seq data in GM12891 et GM19099 treated with TNFalpha) Site studied by luciferase assays in this study.

Commentaire [MR23]: -1609: last nucleotide of Harlequin-int

Commentaire [MR24]: -1512: First nucleotide of the POL2A bound region at exon 1b

Commentaire [MR25]: -1290: Last nucleotide of exon 1b in *UGT2B17_n3*

Commentaire [MR26]: -1217: Last nucleotide of exon 1b in *UGT2B17_n4*
Exon 1b extends to nucleotide -1217 in *UGT2B17_n4* (81 additional nucleotides that are not in UGT2B17_n3)

Commentaire [MR27]: -1064: Last nucleotide of HERVH48-int

Commentaire [MR28]: -983: Last nucleotide of the POL2A bound region at exon 1b

Commentaire [MR29]: -828: Last nucleotide of ATAC-seq peak near exon 1b

Commentaire [MR30]: -902 to -619: CTCF bound region

Commentaire [MR31]: -158 to -147: FOXA1 binding site important for UGT2B17 expression in the liver and prostate.
The single nucleotide polymorphism known to influence hepatic expression of UGT2B17 is underlined.

Commentaire [MR32]: -64: First nucleotide of exon 1 in NM_001077.4 and in *UGT2B17_n2, _n3, _n4* transcripts

Commentaire [MR33]: *UGT2B17_v1* transcript start site according to NM_001077.3 (NCBI RefSeq)

Commentaire [MR34]: +1 to +3: First codon

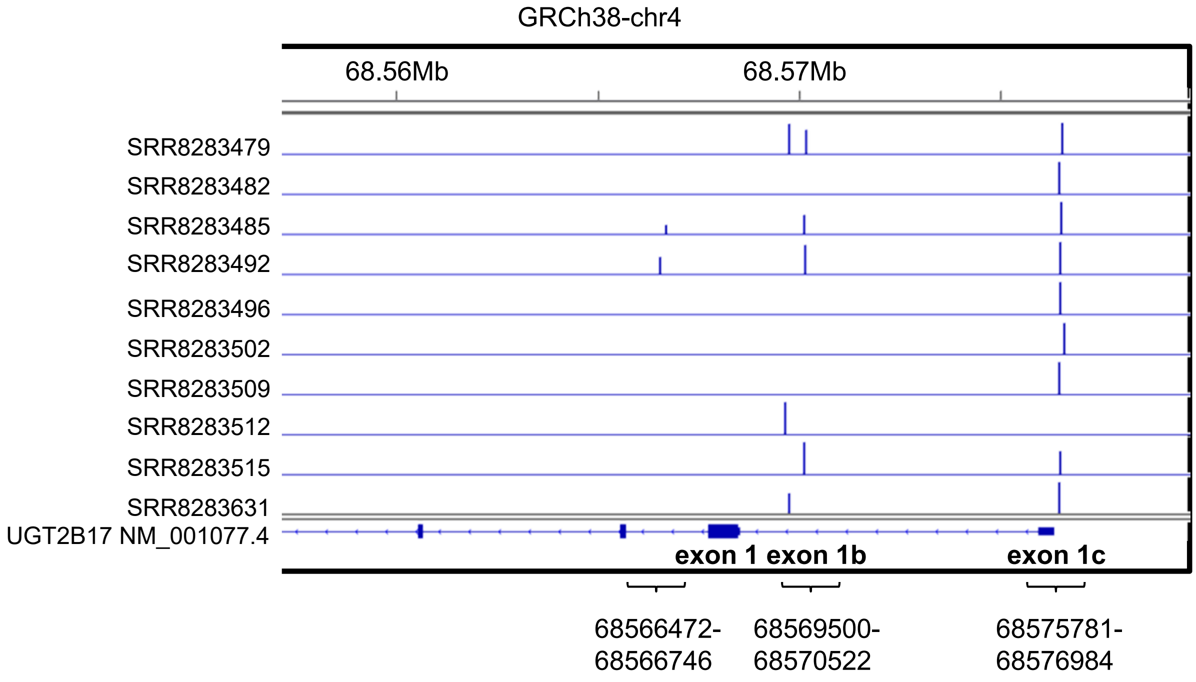

Supplementary Figure 1. ATAC-seq signals at the *UGT2B17* gene locus in CLL cases from a previous study (19). Ten of the 18 CLL samples in this study showed an ATAC-seq signal at the exon 1c and exon 1b of the *UGT2B17* gene locus. Coordinates of ATAC-seq signals on the GRCh38 chromosome 4 are provided. Note that the *UGT2B17* locus is on the reverse strand/orientation.

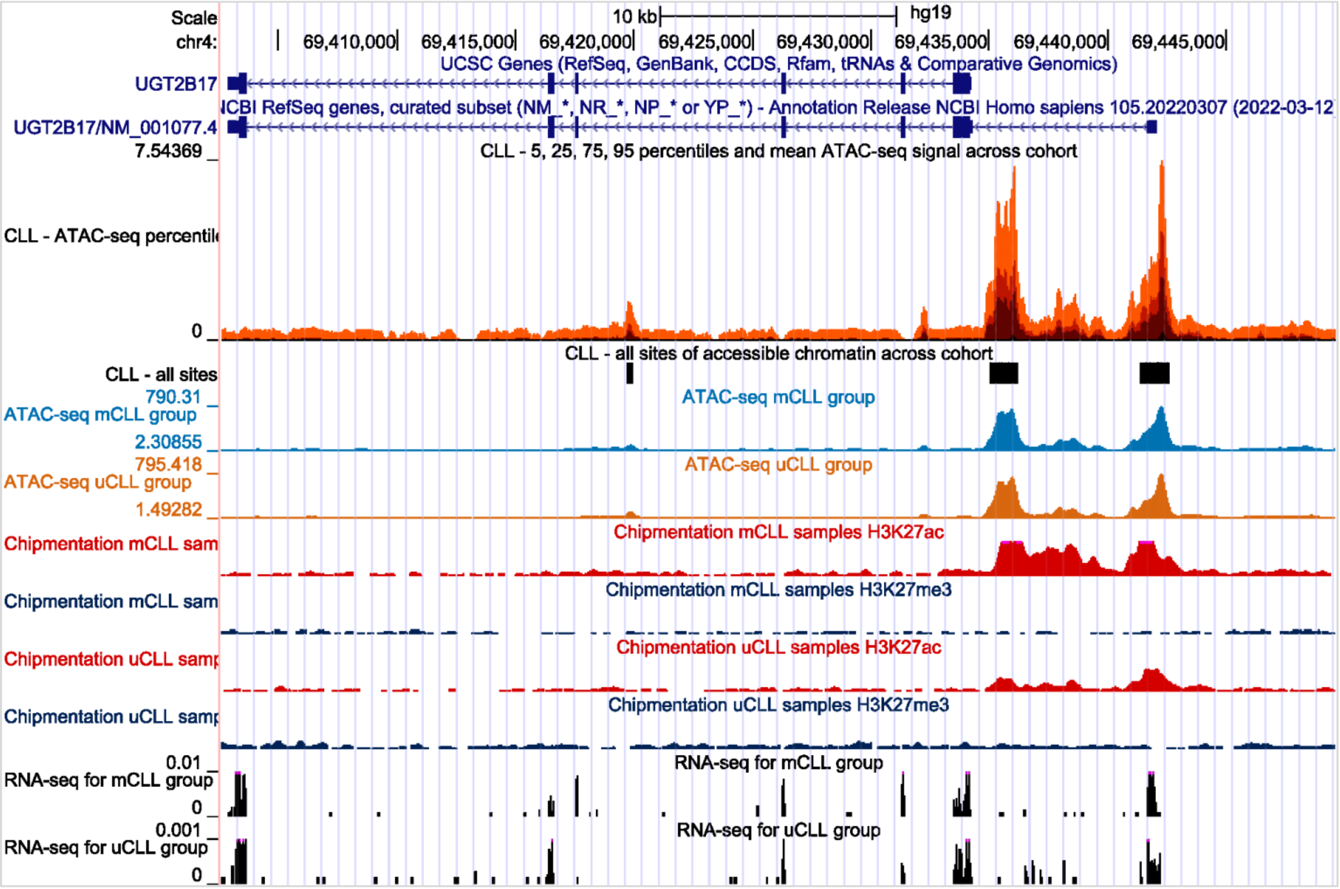

Supplementary Figure 2. ATAC-seq and epigenetic marks at the *UGT2B17* gene locus in CLL cases from a previous study (16). ATAC-seq signals in IGHV mutated (mCLL) and unmutated (uCLL) subgroups of CLL patients are detected near the exons 1c and 1b. Histone H3K27 acetylation (ac) signals, RNA-seq reads and absence of the repressive H3K27me3 signals are consistent with active transcription at exons 1c and 1b. Coordinates of the *UGT2B17* gene locus on the GRCh37 chromosome 4 are provided at the top. Note that the *UGT2B17* locus is on the reverse strand/orientation. Data were obtained from a previous study (16) and accessed on the UCSC Genome Browser as described in Materials and Methods.

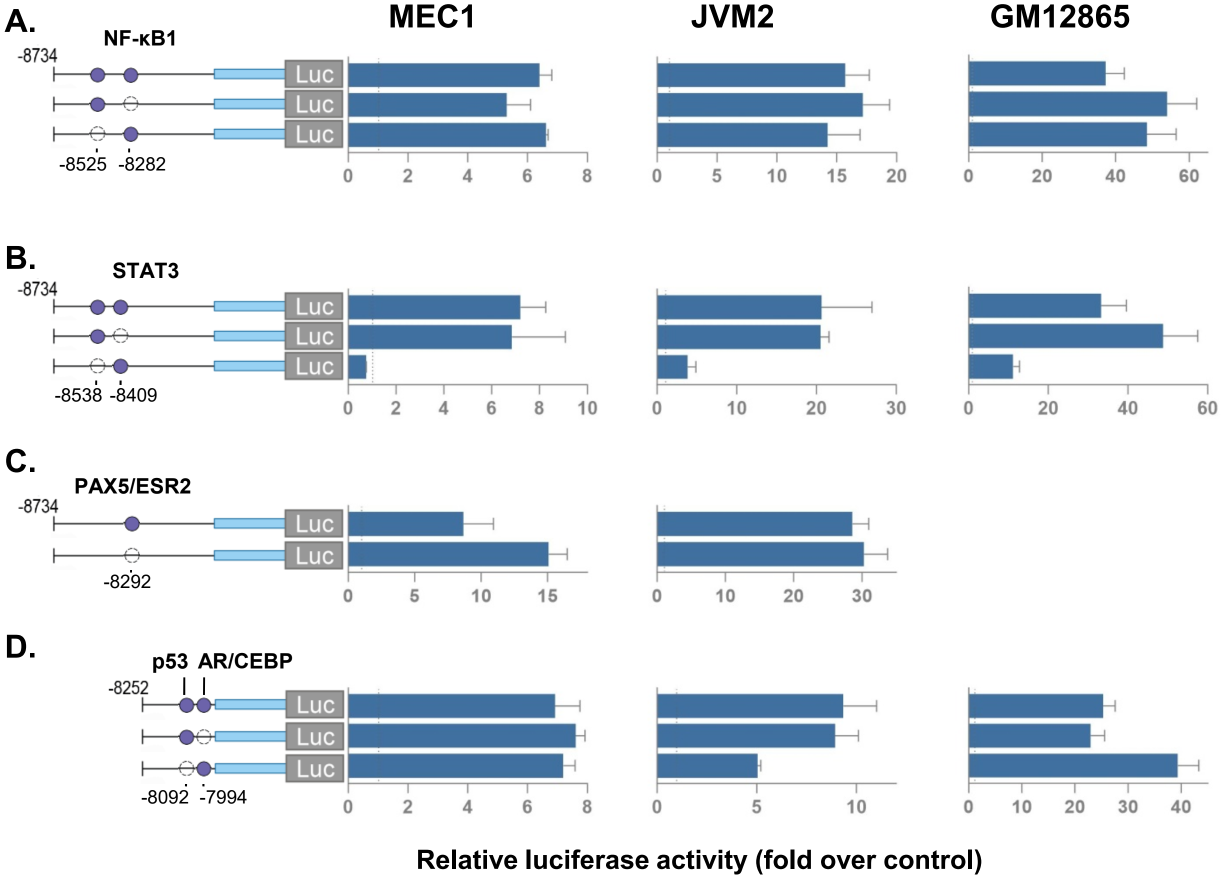

Supplementary Figure 3. Evaluation of active cis-regulatory regions in the P3 promoter of *UGT2B17_n2* transcript by luciferase assays and mutagenesis. Transcription factor (TF) binding sites predicted by JASPAR in the P3 promoter are shown in Figure 2A. Luciferase (Luc) activity driven by the P3 promoter without (filled circles) or with (open circles) mutated TF binding sites is shown relative to the pGL3 empty vector (dashed line). These initial luciferase assays constituted a first screen to identify most active regulatory sequences. Assays were conducted once in triplicates. **A.** NF-κB1: nuclear factor kappa B subunit 1 (p105); **B.** STAT3: Signal transducer and activator of transcription 3. Note that mutagenesis of $STAT3_{-8409}$ also disrupted a BLC6 binding site. The regulatory $STAT3_{-8538}$ binding site studied further in this paper is shown as a positive control. **C.** PAX5: paired box 5. Mutagenesis of PAX5 also disrupted an ESR2 and RORA binding sites. **D.** p53: tumor protein p53; AR: androgen receptor; CEBP: CCAAT/enhancer binding protein. Mutagenesis of p53 also disrupted a IRF6 and ESR2 binding sites, and mutagenesis of CEBP also disrupted the AR binding site. Coordinates are relative to the UGT2B17 translation start site (+1). Representations not drawn to scale.

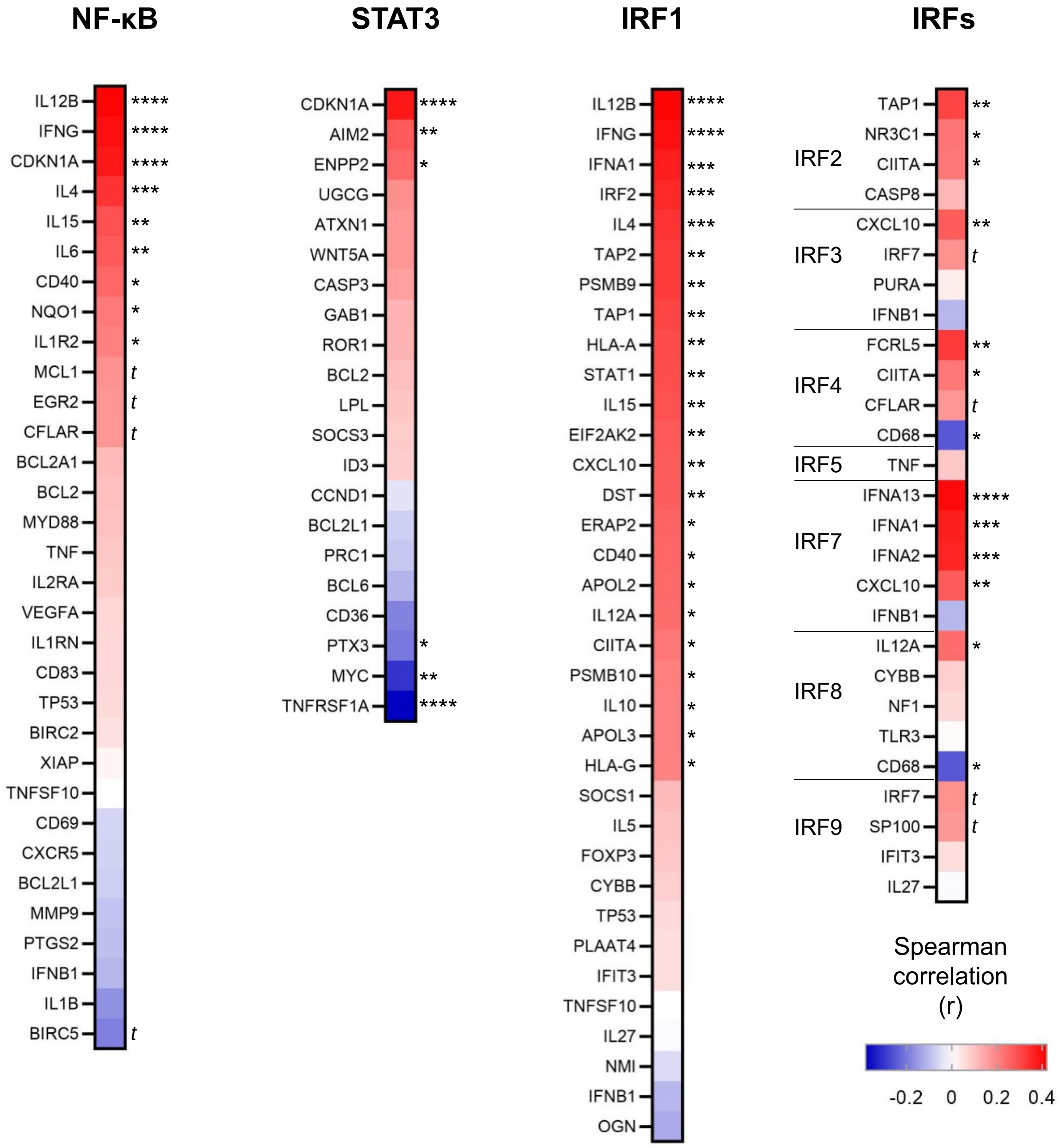

Supplementary Figure 4. Spearman correlations between expression of NF-κB, STAT3 and IRF target genes and *UGT2B17* in the LL100 collection of lymphoma and leukemic cell models. *, *P*≤0.05; **, *P*≤0.01; ***, *P*≤0.001; ****, *P*≤0.0001; *t*, trend.

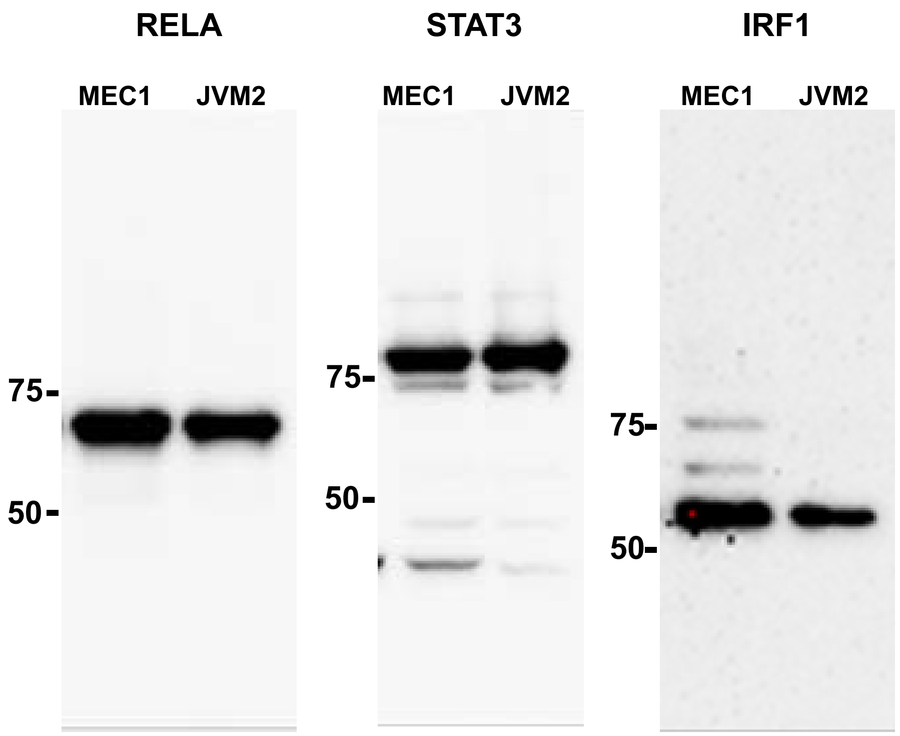

Supplementary Figure 5. Full blot images of immunoblots presented in Figure 3A.

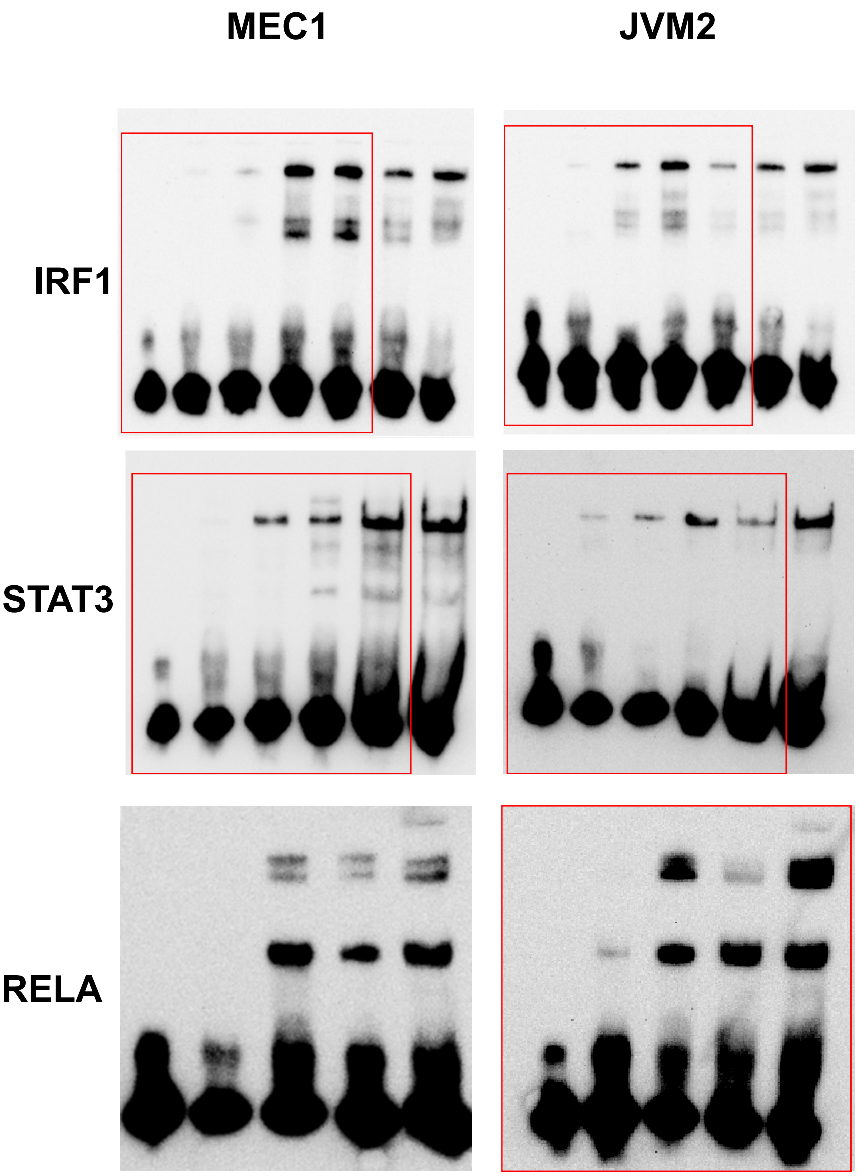

Supplementary Figure 6. Full blot images of EMSA presented in Figure 3B. Images presented are those of each full length membrane. The images shown in Fig.3B are delimited by red squares. Note that the signal for MEC1-RELA (bottom left blot) is complete. It appears incomplete on the lower right side due to a cropped membrane. The large amount of non-shifted probe produced a signal that was beyond the edges of the membrane.

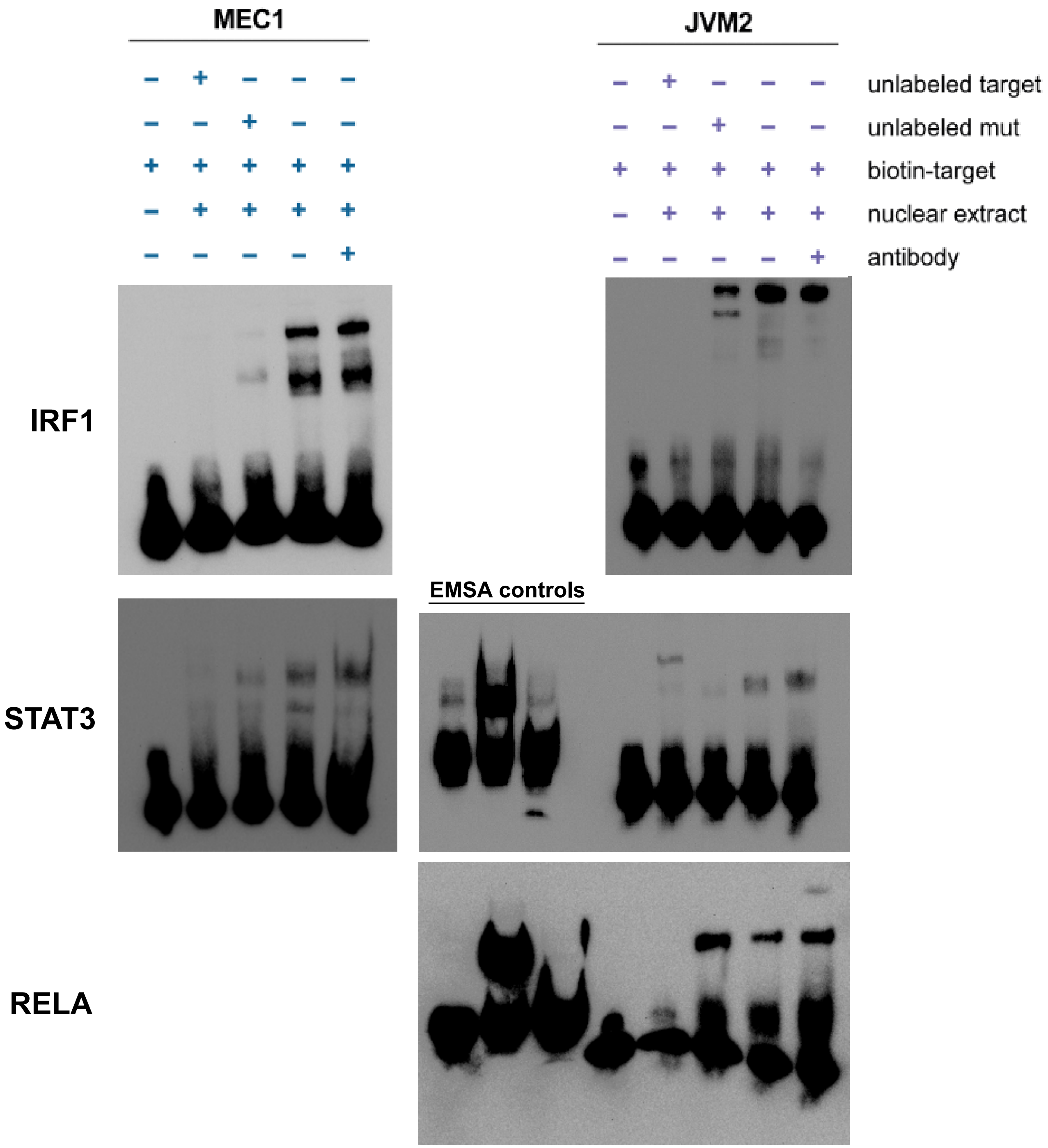

Supplementary Figure 7. Full blot images of replicate EMSA experiments. Images presented are those of each full length membrane. For JVM2 STAT3 and RELA EMSA, the 3 left lanes are control EMSA samples included in the EMSA kit and are unrelated to the MEC1 and JVM2 samples.
